# One species or four? Yes!...and, no. Or, arbitrary assignment of lineages to species obscures the diversification processes of Neotropical fishes

**DOI:** 10.1371/journal.pone.0172349

**Published:** 2017-02-24

**Authors:** Stuart C. Willis

**Affiliations:** Department of Life Sciences, Texas A&M University-Corpus Christi, Corpus Christi, Texas, United States of America; National Cheng Kung University, TAIWAN

## Abstract

Species are fundamental units in many biological disciplines, but there is continuing disagreement as to what species are, how to define them, and even whether the concept is useful. While some of this debate can be attributed to inadequate data and insufficient statistical frameworks in alpha taxonomy, an equal part results from the ambiguity over what species are expected to represent by the many who use them. Here, mtDNA data, microsatellite data, and sequence data from 17 nuclear loci are used in an integrated and quantitative manner to resolve the presence of evolutionary lineages, their contemporary and historical structure, and their correspondence to species, in a species complex of Amazonian peacock “bass” cichlids (*Cichla pinima sensu lato*). Results suggest that the historical narrative for these populations is more complex than can be portrayed by recognizing them as one, two, or four species: their history and contemporary dynamics cannot be unambiguously rendered as discrete units (taxa) at any level without both choosing the supremacy of one delimitation criterion and obscuring the very information that provides insight into the diversification process. This calls into question the utility of species as a rank, term, or concept, and suggests that while biologists may have a reasonable grasp of the structure of evolution, our methods of conveying these insights need updating. The lack of correspondence between evolutionary phenomena and discrete species should serve as a null hypothesis, and researchers should focus on quantifying the diversity in nature at whatever hierarchical level it occurs.

## Introduction

Despite a “renaissance” of species delimitation [1], there is continuing disagreement among evolutionary biologists and systematists as to what species are, how to define them, and even whether the concept is useful to broader goals of biology and conservation [2]. Operationalists argue that for a concept of species to be useful, the concept must define both what species are and how to recognize them (e.g. [3]). Under this philosophy, failure to meet species criteria precludes recognition of a group as a species (or at the rank of species). In contrast are researchers who see “species” as an ontological concept wholly separate from species’ “contingent properties” and who emphasize that traits used by operationalists may arise in different orders if at all during speciation [4, 5]. Importantly, this viewpoint portrays the tips of the tree of life as a discrete series of units, albeit ones challenging to delimit, but does not question how well this dichotomy reflects biological reality [4]. Nevertheless, many authors thus argue that multiple, integrated datasets should be used to recognize species [6, 7].

Still another group of biologists contend that applying “species” is counterproductive, since the dynamic and multifaceted nature of evolution is unlikely to produce the kinds of discrete units implied by the various conceptualizations of species. As such, “species” may actually be holding back progress in understanding and conserving biological diversity [8, 9]. These biologists emphasize the processes by which biological diversity is created, rather than on arbitrary classification of a continuum of hierarchical diversity. Indeed, Darwin himself wrote about the confusion surrounding “species”, that “It all comes, I believe, from trying to define the indefinable” [10], and that “I look at the term species, as one arbitrarily given for the sake of convenience to a set of individuals closely resembling each other” [11]. In the *Origin of Species*, Darwin emphasized the development of differences among “local varieties” or “races” as the units of evolution, using “species” as a convenient bridge with a familiar terminology used by Linnean taxonomy [12]. However, many now agree this terminological holdover has generated more heat than light in understanding patterns and processes of diversification [13].

In addition to the confusion inherent in “species”, there are logistical difficulties to objectively documenting units under this philosophy. In most cases, species in sympatry are *relatively* easy to distinguish as units that subdivide ecological resources, do not exhibit a common gene pool, and are often morphologically discrete (in this sense, these are synonymous with “populations” in the sense used by community ecologists; [14]). However, in considering demes that comprise geographically widespread series of similar individuals, delimitation is often more challenging. On the one hand, a degree of phenotypic (morphological, ecological) divergence is expected among sub-populations due to genetic drift and local adaptation [5, 15], whereas the rate and efficacy of the metaphorical glue that holds populations together, gene flow, is impossible to observe in real time [16, 17]. To address this, some methods provide a statistical perspective on whether observed morphological differences between hypothesized species are sufficiently discontinuous to warrant recognition, taking into account sample size and within group variability [15, 18]. Moreover, many delimitations of species now incorporate ecological and genetic data that bring to light some of the process and nuance of population structure and divergence exhibited by the products of evolution [7, 19–21].

The use of diverse datasets and methods to characterize evolutionary lineages is especially critical in highly diverse tropical regions where meta-populations are often widespread and exist in proximity to many closely related lineages. However, one of the domains where complementary analytical frameworks have yet to see widespread use is in alpha taxonomy of the most species dense assemblage of vertebrates on Earth, freshwater fishes of tropical South and Central America (the Neotropics). The Neotropics harbor over half of the freshwater fish species on Earth, and one tenth of all vertebrate species [22, 23], but the forces responsible for this are not well understood, and likely include an interacting retinue of historical biogeography, biological interactions, habitat heterogeneity, and relative climatic stability [24]. Recent predictions based on the pace of species description place the estimate of Neotropical freshwater fish species at over 8,000, a success attributed in part to the continued training of fish taxonomists [25], a group that is also under pressure to document diversity for the sake of conservation in the face of rapid anthropogenic habitat alteration across the tropics [26, 27]. However, alpha taxonomy of Neotropical freshwater fishes continues to rely largely on meristic morphological data, small sample sizes relative to the geographic distribution of these lineages, and limited statistical testing.

One of the economically, and ecologically important groups of Neotropical fishes that aptly illustrate this pattern is the peacock “bass” cichlids of the genus *Cichla* (Schneider, 1801). *Cichla* are large, colorful predators endemic to floodplain rivers of tropical South America east of the Andes [28]. In 2006, Kullander and Ferreira (hereafter K&F) revised the taxonomy of *Cichla*, describing 9 new species and resurrecting a 10^th^, bringing the current number to 15 [29]. Like most reviews of Neotropical fish taxonomy, this revision used morphological data with no statistical framework, applied meristic central tendencies and ontogenetically and geographically variable color characteristics to distinguish species, and included a diagnostic key that relied heavily on geographic origin. Around the same time, colleagues and I began making extensive tissue collections to estimate species boundaries in *Cichla* using molecular data [30]. In our recent work [31], we examined the correspondence of combined molecular data from over a thousand individuals with the 15 species identified by K&F, who cited a phylogenetic species concept [32]. We also inferred species under a polytypic species concept, one that provides for limited incomplete reproductive isolation between species but that refers populations that intergrade to sub-specific units [33]. Some of the species recognized by K&F corresponded to well-circumscribed molecular clusters, and these also tended to be distinguishable by multiple morphological characters. Other species graded together or were completely indistinguishable with molecular data, and were those diagnosed mostly on the basis of allopatric distributions, overlapping meristics, and globally variable color characters (see S1 Fig).

**Fig 1:**
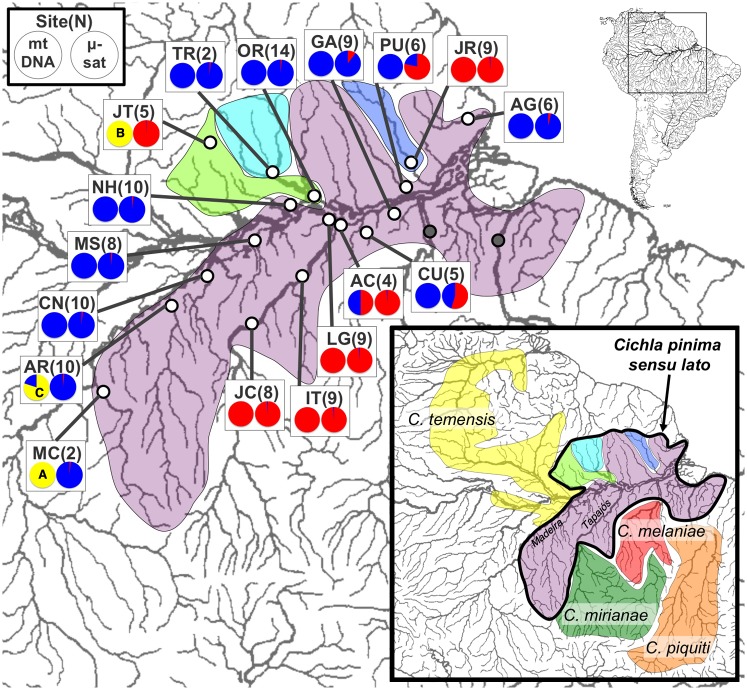
Map of sampling sites and the distribution of mitochondrial DNA clades (from Fig 2) and microsatellite clusters (K* = 2; Fig 4) for *Cichla pinima sensu lato*. The pie chart for each locality shows the proportion of individuals bearing The inset shows distributions of all of the species of *Cichla* clade A.

Despite the application of species concepts that provide for multi-faceted evolutionary lineages, one set of individuals eluded definition even under these broad ontologies. Four clade A species (sensu [30]) described by K&F from the eastern Amazon, *Cichla pinima*, *C*. *vazzoleri*, *C*. *jariina*, and *C*. *thyrorus*, are more morphologically similar to each other than to the other species of clade A, exhibit geographically contiguous ranges, and were defined based on highly variable and overlapping coloration patterns and meristics. Whereas molecular data showed two very strong clusters among these individuals, including two non-sister mtDNA lineages, neither of these clusters corresponded to any of the described species (Fig 1). Moreover, while there appeared to be admixture between these two clusters in several locations, the distribution of each cluster was not one that suggested simple isolation by distance, hybridization, or incomplete lineage sorting, as we observed admixed and non-admixed localities from both clusters haphazardly distributed east (downstream) of the Tapajós River mouth. In contrast to the polyphyletic mtDNA, the concatenated nuclear sequences from 21 nuclear loci portrayed all representatives of these species to be monophyletic [34], and we hypothesized that this meta-population represented a persistent ancestor or hybrid species, and refer to it collectively as *C*. *pinima sensu lato*. However, the summary of these individuals as a single species may synonymize two non-monophyletic lineages and obscures a significant and complex population structure, evolutionary history, and ongoing diversification, so here I analyze these data together in an integrated and quantitative manner to understand the processes that gave rise to this complex pattern and better resolve the species lineages of *Cichla*. However, the findings challenge current practices for recognizing species, so I briefly critique the application of “species” for biodiversity communication and conservation using Neotropical freshwater fishes as an example.

## Methods

Molecular data were generated in previous studies [31, 34]; please see these references for Genbank and Dryad accession numbers. Data are described in Table 1 and S1 and S2 Tables. Below is a brief narrative of the analyses utilized here; for additional analysis parameters, see S1 File.

**Table 1:** Sample sizes per locality of *Cichla pinima sensu lato* for mitochondrial DNA, 11 microsatellite loci, and sequences from 2 more variable and 15 remaining nuclear loci.

Code	Locality	mtDNA/microsats				nuclear: 2 loci/15 loci			
		*pin*^a^	*vaz*	*thy*	*jat*	*pin*	*vaz*	*thy*	*jat*
JT	Jatapu (Uatumã)		5/5				5/1		
MC	Machado	2/2				1/0			
AR	Aripuanã	13/10				2/1			
CN	Canumã	10/10				1/1			
MS	Maués	9/8				0			
NH	Nhamunda	10/10				0			
TR	Trombetas (abv. rapids)			2/2				2/2	
OR	Oriximiná		15/14				2/1		
JC	Jacareacanga	8/8				0			
IT	Itaituba	10/9				3/1			
LG	Lago Grande	9/9				0			
AC	Alter do Chau	4/4				2/1			
CU	Curuá-Una	5/5				1/1			
PU	Paru	6/6				1/1^b^			
GA	Guajara	10/9							
JR	Jari (above waterfalls)				9/9				5/2
AG	Araguari	6/6				1/1			

^a^*pin*: *Cichla pinima sensu stricto*; *vaz*: *C*. *vazzoleri*; *thy*: *C*. *thyrorus*; *jat*: *C*. *jatapu*;

^b^missing locus 1835e6

## MtDNA phylogeography

Two analyses of mtDNA data were made to provide a preliminary foundation for the investigation. First, an mtDNA genealogy of *Cichla* was inferred under a relaxed molecular clock in the program Beast 2.4 [35] to test the hypothesis that topological results in our previous analyses (described in the Introduction) derived from improper branch length estimation due to the application of an additive, but not ultrametric, maximum-likelihood algorithm [31]. The analysis here used haplotypes from the control region (CR), as well as ATPase 8,6 gene (ATP) sequences from a subset of individuals (121 of 324 CR haplotypes). To calibrate coalescence times, a mutation rate was applied that was derived from comparison with cytochrome *b* data for these species [30, 36], and is consistent with the long term mutation rate (>1mya) in African cichlids [37].

To infer the geographic and temporal origin of the distribution of the mtDNA lineages observed in *Cichla pinima sensu lato* and test the hypothesis that the distribution of these mtDNA lineages has been historically stable, spherical phylogeography analysis was performed with Beast [38, 39]. This analysis takes the geographic coordinates of each tip in the genealogy and infers the distribution of the ancestors while accounting for uncertainty in phylogenetic reconstruction. This analysis only included mtDNA data from *C*. *pinima sensu lato* since the tips in this analysis are alleles (gene copies) and not haplotypes, and the Markov chain was slow to mix with just these data. Two localities of *C*. *pinima sensu lato* were excluded, the lower Xingu and Tocantins, that exhibit introgressed DNA from non-*C*. *pinima sensu lato* species (see Results).

### Population structure with microsatellites

To test the degree to which the demes (groups of individuals with approximately random mating, here taken to be localities; see [37]) of *Cichla pinima sensu lato* form a single population and provide an estimate of the pattern and magnitude of gene exchange among them, two F_ST_ analogs were calculated. The first, an AMOVA-based F_ST_, considered only the identity of alleles, calculated in Arlequin 3.5 [40]. This analysis used 11 of the 12 loci from [31], excluding one locus due to inconsistent amplification (null alleles). Genetic distances were also inferred using the distance between alleles (R_ST_; [41]), also in Arlequin. Here, 9 of the 11 loci were used because 2 exhibited single-base mutations in di-nucleotide motifs (S1 Table).

Individual clustering, which does not assume that each locality is a homogenous deme, was applied to further test the hypothesis of population structure using Structure v.2.3.4 [42]. Structure fits individuals into a pre-defined number of clusters (K), and the optimal number of clusters (K*) can be determined by observing the increases in variance among replicate runs as K increases (ΔK) [43]. Structure was run and the mean likelihood and second order rate of change (ΔK) calculated with K from 1 to 10 to determine K* for both the 11 locus and 9 locus datasets.

### Testing species hypotheses with sequence data and species trees

One of the hallmarks of species is that they have a separate evolutionary trajectory, that is, a projection of evolving separately based on a history of doing so (sensu the “evolutionary species concept”, [4, 44, 45]). This phylogenetic structure can be modeled by the multi-species coalescent (MSC) [46], a model that provides for the deep coalescence (incomplete lineage sorting) of gene lineages between species but generally not for gene flow between lineages after divergence. The MSC model provides a statistical framework to test hypotheses of separate species lineages using gene trees from neutral molecular markers, and thus has been suggested to be more objective than traditional morphological taxonomy [47]. One implementation of MSC-based delimitation calculates the Bayes Factors between species tree models with different arrangements of species as a way to compare their fit to the evolutionary concept of species (Bayes Factors Delimitation, or BFD, [48]; see also [49, 50]). Here, Bayes Factors were calculated from the marginal likelihood of the species tree with different arrangements of individuals into hypothetical species using the *Beast MSC model and path sampling in Beast 2.4 [51, 52]. Species hypotheses consisted of one species, the four described species, and divisions reflected in the phylogeographic and population structure results. This analysis used haplotypes from 17 of the 21 nuclear loci from our previous analysis [34], which consist of introns and anonymous loci, in addition to mtDNA (ATP) data (S2 Table). Analyses were run with several dataset variations: with and without mtDNA, and with mtDNA but without data from localities downstream of the Tapajós River mouth or Lago Grande (LG). To quantify the expectations of gene tree-species tree discordance among lineages of clade A, the BFD-optimal species delimitation hypothesis was used as a framework to estimate coalescent branch lengths using the program BPP [53].

A second fit of the multi-species coalescent for species delimitation is the package STACEY [54], also in Beast. One limitation to the BFD analysis is that only a limited number of hypotheses can be tested, and which depend on robust inference of terminal units [55, 56]. However, in STACEY analyses, terminal units are generally demes (e.g. localities), not hypothetical species, and a species tree is inferred among these tips with a birth-death-collapse tree prior in which branches shorter than a specified length (collapse height) are collapsed to a single branch tantamount to a species. This allows the sequence data themselves to guide the species hypotheses tested, without the limitation of *a priori* hypotheses. Four arrangements of the data were made to further test conformation of *Cichla pinima sensu lato* populations with the evolutionary concept of species: with and without mtDNA, and with and without the downstream localities. OTUs in this analysis were localities of *Cichla pinima sensu lato* for which data from all loci were available; other species were coded as delimited by [31]. Analyses were also performed that included the Machado (MC) locality, for which only data from the mtDNA and two nuclear loci were available. Several iterations of the first dataset with different values for the STACEY priors were also run to test their effects.

### Corroborating species hypotheses with population patterns

The species tree delimitation analyses provide statistical support for hypotheses based on the mtDNA and microsatellite data, but provide limited quantitative reconciliation of the population-level data with these lineages. To test the power of these hypothesized lineages to explain contemporary population structure, several further analyses of the microsatellite data were performed. First, analysis of molecular variance (AMOVA, [57]) was performed on the 11 and 9-locus microsatellite datasets in Arlequin against the hypothesized species arrangements and several further divisions reflecting observed population structure. Next, the assignment probability and mixture proportions for the downstream and Madeira tributary localities was tested using *a priori* assignment of core localities from the hypothesized species lineages to pre-defined clusters in Structure. This analysis was repeated for several of the well-supported species hypotheses, using different *a priori* assignments of samples. Finally, historical hypotheses for how the downstream and lower Madeira localities could have come to show their contemporary patterns were tested by estimating the probability of different scenarios of population history using approximate Bayesian computation in DIYABC [58]. These models tested whether scenarios of pure divergence were a better explanation of the data than admixture (S3 and S4 Figs), and their posterior probabilities of each scenario were estimated via simulation from diffuse priors. This analysis used the 9-locus microsatellite dataset under a generalized stepwise mutation model (stepwise with some larger, multi-step mutations). Population scenarios for the downstream and Madeira tributary samples were simulated separately.

## Results

**Fig 2:**
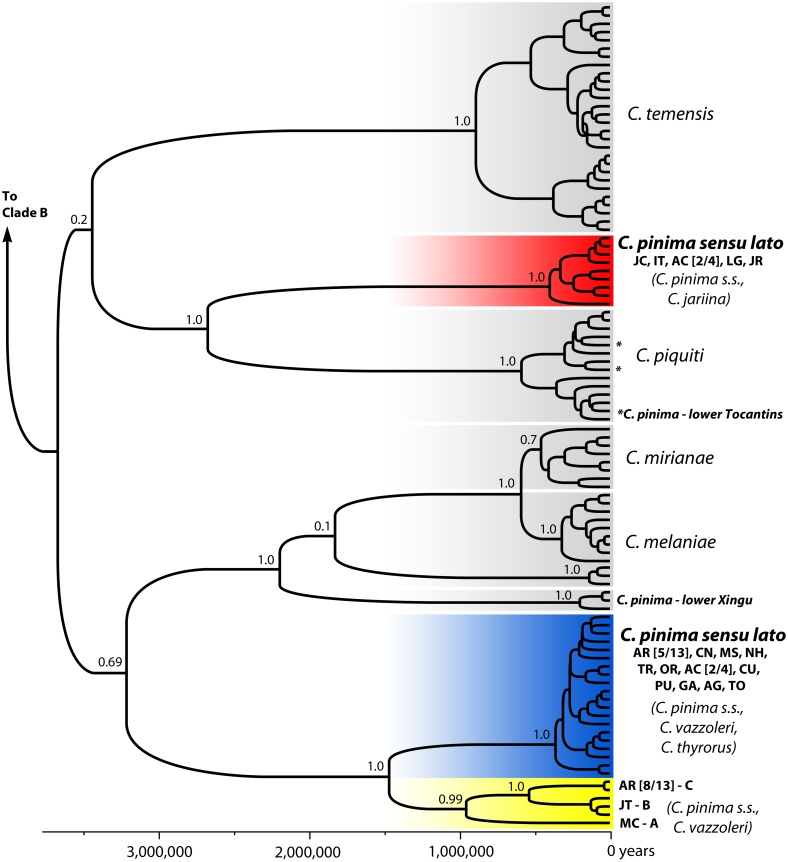
Mitochondrial DNA genealogy of *Cichla* clade A. Node values are posterior clade probability. For *Cichla pinima sensu lato* (indicated in bold), the localities where those clades were observed, as well which described species (*C*. *pinima sensu stricto*, *C*. *jariina*, *C*. *vazzoleri*, *C*. *thyrorus*) were observed in each clade, are denoted. Locality codes follow Table 1 and Fig 1, and the values associated with some localities are (the number of individuals bearing haplotypes from that clade / total individuals collected at that locality). For the endemic western lineages (yellow), each is labeled to match the mtDNA reference in Fig 4 (A,B,C). A fully annotated version of this genealogy is presented as S1 Fig.

There were no substantive differences between the mtDNA genealogy of all *Cichla* inferred here (Fig 2, S2 Fig) and prior results from these data [31], indicating that the topology of this genealogy, including the mtDNA polyphyly of the four focal species, does not result from improper branch length estimation. Within clade A, individuals assigned to *C*. *pinima sensu lato* exhibited haplotypes in four mtDNA lineages which did not correspond to the four species described by K&F. All of the haplotypes from three of these four species (*C*. *jariina*, *C*. *vazzoleri*, *C*. *thyrorus*) were found mixed in the two more common mtDNA lineages, while the remaining lineages were observed in individuals assigned to *C*. *pinima sensu stricto*. One of the mtDNA lineages of *C*. *pinima s*.*s*. reflected haplotypes nested among a lineage otherwise found only in *C*. *piquiti*, and were found only in the lower Tocantins adjacent to *C*. *piquiti*. Another lineage grouped with the mtDNA lineages of *C*. *melaniae* and *C*. *mirianae*, and was found only in the lower Xingu proximal to *C*. *melaniae*. These lineages are hypothesized to result from recent or ancient introgression [31], and were not analyzed further here (but see Discussion). The remaining two lineages were not sister in the genealogy, but were both widespread throughout *C*. *pinima sensu lato* (Figs 1 and 2). One of these lineages (“southern”) was most common in the Tapajós River (the southern “core”) and an area adjacent to it (Lago Grande, LG), but also found in the Jari River (*C*. *jariina*), an Amazonas tributary downstream of the Tapajós. The other was the only mtDNA lineage in the western range (“western”), but was also found in most localities downstream of the Tapajós, as well as in the Tapajós mouth (AC) alongside the southern lineage (2 of 4 individuals). Within the western range, most localities (CN, MS, NH, TR, OR) presented haplotypes of a diverse sub-clade with no obvious substructure (the western “core”), and it was this clade that was most-common downstream of the Tapajós. However, the Jatapu (JT) in the northwest and the Machado (MC) and Aripuanã (AR) in the southwest, each exhibited unique mtDNA lineages related first to each other, then sister to the widespread western sub-clade. Intriguingly, the Aripuanã also exhibited haplotypes from the more common western clade (5 of 13 individuals). The more common western clade was also found in one of four *C*. *pinima* in the lower Tocantins (the other three bore *C*. *piquiti* haplotypes).

Spherical phylogeography rejected the hypothesis that the distribution of mtDNA clades has been stable, and instead showed that the two main lineages only recently came to occupy the Amazon downstream of the Tapajós. This analysis portrayed the two main mtDNA clades of *C*. *pinima sensu lato* as being allopatric for the majority of their evolution, with ancestral areas in the middle Tapajós (IT and JC) and lower Madeira Rivers (AR and CN) (S5 Fig). The location of the root of this genealogy was not estimated because these two lineages are not sisters. During this period of allopatry, the analysis reconstructed the western clade as first dispersing north to the mainstem Amazon and smaller tributaries (MS, TR, OR, NH; the lineage “core”), followed later by dispersals south to the Machado (MC) and north again to the Jatapu (JT). Finally, only recently (compared to the total time sampled by the genealogy) and nearly simultaneously, both the western and southern lineages expanded from allopatry to jointly colonize the eastern Amazonas basin. Based on calibration of the full genealogy (μ = 0.02 mut./site/my), the most recent common ancestors of these sub-clades that now occupy the lower Amazonas had median ages of ~395 thousand years ago, tya (95%HPD 220–580 tya) and ~345 tya (206–530 tya) for the southern and western lineages, respectively. In contrast, the divergence of the Jatapu/Madeira lineages from haplotypes in the Western core and lower Amazonas was estimated at ~1.46 million years ago, mya (1.06–1.96 mya). While the mutation rate used here is consistent with calibrations from other cichlids [59], it should be noted that because of the time-dependence of mutation rates [60], the absolute divergence times of these lineages should be interpreted with caution; however, the relative divergence times are expected to remain the same.

**Fig 3:**
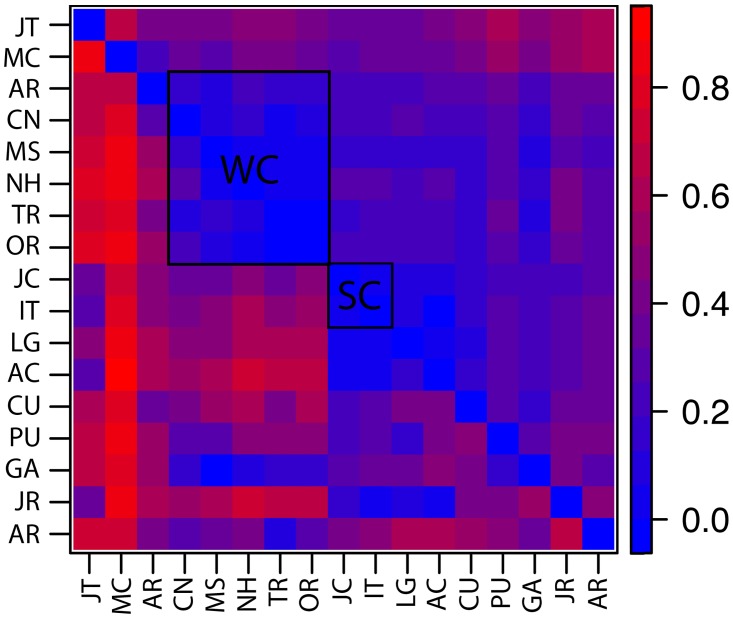
Heat map for genetic distances between sampling localities of *Cichla pinima sensu lato*. Below diagonal, F_ST_ (11 loci); above diagonal, R_ST_ (9 loci), after S3 Table. The “core” regions are indicated: “WC”, western core; “SC”, southern core.

Genetic distances from microsatellite data portrayed most of the localities of *C*. *pinima sensu lato* as being significantly divergent from one another, with the exception of localities with small sample sizes (Fig 3, S3 Table). Size-based distances (R_ST_) were generally larger than identity-based ones (F_ST_), and permutation with Spagedi 1.4b [61] confirmed that allele size explained significant population structure (multi-locus p<0.001). The magnitude of genetic distances (mean/max R_ST_ 0.45/0.89) reflected restricted gene flow among most localities, although there appeared to be less restricted gene flow within the western (0.11/0.27) and southern (0.06/0.13) core regions. This structure can best be described as a wide-spread meta-population structure, with greater sub-structure among localities outside of the two core regions.

**Fig 4:**
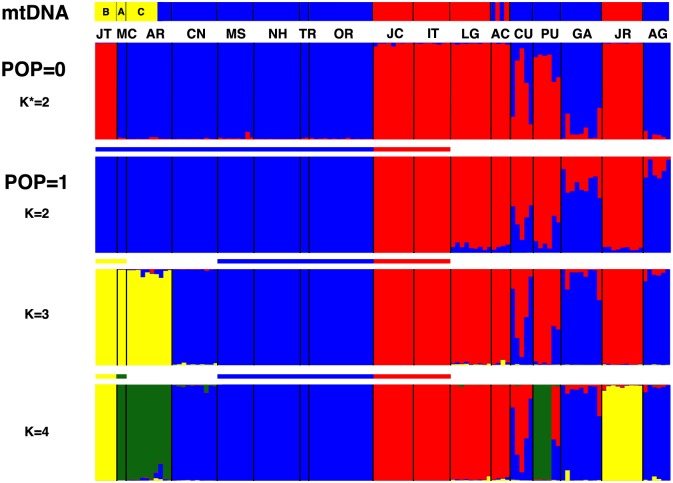
Structure results for 11 microsatellite loci from *Cichla pinima sensu lato*. Pop = 0, no sample pre-assignment; Pop = 1, some samples pre-assigned to K clusters, indicated by the bars above the Structure plots. The distribution of mtDNA clades (after Fig 1) is provided for reference. Locality codes follow Table 1 and Fig 1.

Two clusters were optimal both for 11 and 9 loci based on ΔK (Fig 4, S6 and S7 Figs), reflecting a broader geographic structure than portrayed by the F_ST_ analogs, but one that was consistent with the higher gene flow within the core regions (Table 2). These clusters largely corresponded to the distribution of the two mtDNA clades also (Fig 1) except that the Japatu (JT) fishes clustered with the Tapajós (“southern”) fish. Given the F_ST_/R_ST_ comparisons, the Jatapu-Tapajós clustering suggests a degree of allelic homoplasy perhaps exacerbated by small sample size. Importantly, several localities in the downstream region, in particular Curuá-Una (CU), Paru (PU), and Guajará (GA), exhibited admixture between the two clusters. However, Alter do Chau (AC), which exhibited both southern and western mtDNA lineages, did not. Similar population structuring was portrayed by analysis with the program Structurama [62], though without the admixture option (see [31]), this analysis emphasized more but less consistent clusters of individuals (S4 Table). Runs of Structure with the K* from Structurama confirmed overlap between the clusters (not shown; available upon request).

**Table 2:** Results from *Beast runs with different species and dataset arrangements for Bayes Factor Delimitation of *Cichla pinima sensu lato*.

Bayes Factor Delimitation		All samples				Upstream	
		Nuclear + mitochondrial		Nuclear only		Nuclear + mitochondrial	
Species	Arrangement	log_e_L	BF*	log_e_L	BF	log_e_L	BF
1sp	*pinima s*.*lato*	-20955.6	201.2	-18219.1	141.9	-20459.0	167.3
4sp	as described	-20897.1	84.2	-18171.5	46.7	na	na
2sp	West/South	-20894.6	79.1	-18171.2	46.1	-20393.7	36.9
3sp	W/S/*jariina*	-20885.2	60.3	-18158.8	21.4	na	na
3sp	W/S/Jatapu (JT)	-20868.7	27.4	-18153.9	11.6	-20381.9	13.1
3sp	W/S/(JT+MC+AR)	-20867.3	24.5	-18165.0	33.8	-20388.9	27.2
4sp	W/S/JT/(MC+AR)	-20855.0	**0.0**	-18148.1	**0.0**	-20375.3	**0.0**

*Bayes Factors calculated as 2*(log_e_M_1_-log_e_M_0_).

**Fig 5:**
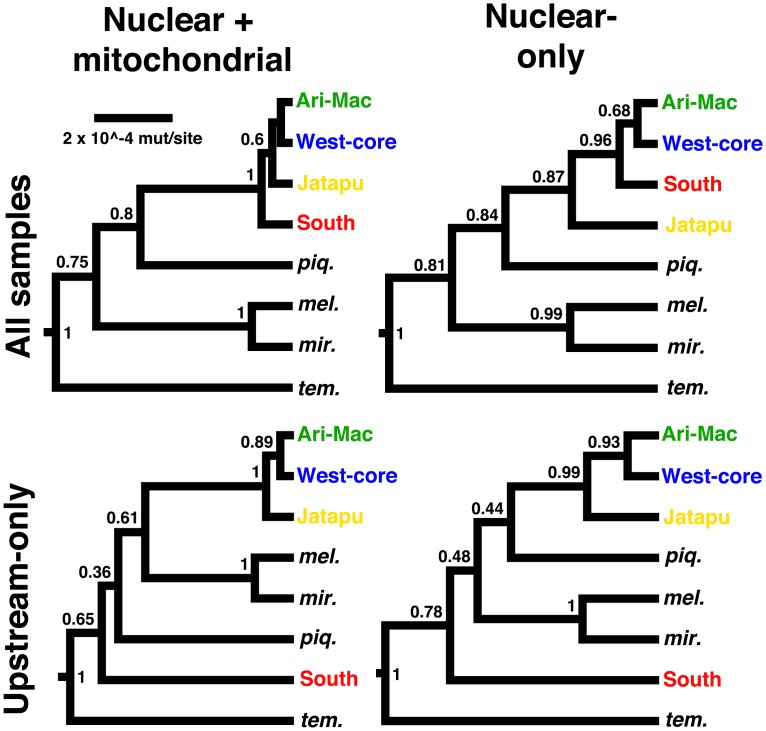
Species trees for each dataset arrangement under the optimal, four-species model from Bayes Factor Delimitation (BFD) of *Cichla pinima sensu lato*. Note: the no mtDNA-upstream only dataset was utilized with the same model but not subjected to path sampling analysis (see Table 2).

Based on the above results, the southern and western populations were hypothesized as separate species lineages, with a likely zone of admixture downstream of the Tapajós, and potential nascent lineages in the Jatapu (JT) and Madeira tributaries (AR, MC). Bayes factors for different species tree models (BFD) portrayed the four described species as being an improvement over a single species, presumably because it provided some flexibility for the deeply divergent gene lineages, but all other hypotheses were superior to these described species (Table 2). Bayes factors also suggested that there was “very strong” evidence (BF >10, sensu [63, 64]) that four species, a southern plus three western lineages, was the best explanation for the data. In contrast, separation of the Jari locality (*C*. *jariina*) provided a smaller increase in likelihood over two lineages. However, the largest improvement in likelihood over one species was the division into southern vs. western, with smaller increases for separation of the Jatapu (JT) and Aripuanã/Machado (AR/MC) lineages, respectively, especially when the downstream data were excluded. Intriguingly, superiority of the new four species model was consistent whether or not the downstream localities were included in analysis, but when the downstream localities were excluded, the southern lineage was no longer monophyletic with the western lineages (similar to the mtDNA genealogy). This suggests that the downstream localities provide a mixed phylogenetic signal, and importantly, this signal is still present when mtDNA data are excluded (Fig 5). Using the optimal BFD hypothesis as a framework in the program BPP [53], the depth of clade A was estimated at ~4 coalescent units (N_E_ generations) [65], suggesting moderate expectations for gene tree-species tree discordance among clade A lineages, but not to the extent that would produce this dual resolution of lineages [66].

The analysis of sequence data with STACEY revealed similar species delimitation results to the BFD analysis, though the optimal solution was not entirely clear (Table 3). The birth-death-collapse model of STACEY was relatively insensitive to changes in the collapseWeight or popScale priors, but was more sensitive to the specified collapseHeight, particularly the one used for summarizing the data. Not surprisingly, smaller values preserved more lineages, and larger ones produced fewer (S5 Table). Between the different datasets and collapseHeights used for summarization, all the terminal units (localities) of *Cichla pinima sensu lato* used here were either retained as species or collapsed into a single species (Table 3). In the latter case, two other species were collapsed to one as well, *Cichla mirianae* and *C*. *melaniae*. These two species were among those corroborated in our previous delimitation analysis, and they exhibit well-defined morphologies and occupy dissimilar habitats [31] (S1 Fig). Because they are also the youngest, relatively unambiguous species pair in *Cichla*, they provide a useful threshold with which to compare the *C*. *pinima sensu lato* results. In both of the analyses using the downstream localities (with/without mtDNA), the deepest node corresponding to *C*. *pinima sensu lato* was on par with, or younger than, *C*. *melaniae*/*C*. *mirianae* (Table 3, see also Fig 5). In contrast, when the downstream localities were excluded, the southern lineage moved deeper in the tree as above, and was retained by the analysis at both collapseHeights (Table 3). Excluding the southern lineage, the deepest branch among the remaining (western) localities of *C*. *pinima sensu lato* was similar to *C*. *melaniae*/*C*. *mirianae*. This makes the STACEY results unambiguously consistent with the BFD support for two lineages (“South” and “West”), but with less clear support for the three western lineages in the optimal BFD model (Table 2). Interestingly, with mtDNA included, the relationships among tips representing the three western lineages were not well supported (PP<0.4). However, when mtDNA was excluded, the Jatapu lineage was supported as diverging first with higher confidence (PP>0.92), suggesting that the close relationship of Jatapu and Aripuanã in the mtDNA tree is in conflict with the nuclear loci.

**Table 3:** Results from runs of STACEY with different data arrangements and different collapseHeight summary values including the Machado locality.

Data^a^		summary collapseHeight: 1x10^-5^			summary collapseHeight: 1x10^-4^			node depths (mut/site)^e^		
		retained^b^	PP^c^	tip arrangements^d^	retained^b^	PP^c^	tip arrangements^d^	mel-mir	west-west	south-west
all-mt+nuc	a	10	0.25	(OR,TR),(AR),(MC),(CN),(JR),(PU),(AG),(CU),(AC,IT),(JT),(mel),(mir)	1	0.51	(OR,TR,AR,MC,CN,JR,PU,AG,CU,AC,IT,JT),(mel,mir)	0.000085	0.000062	0.000085
all-mt+nuc	b	10	0.23	(OR,TR),(AR),(MC),(CN),(JR),(PU),(AG),(CU),(AC,IT),(JT),(mel),(mir)	1	0.52	(OR,TR,AR,MC,CN,JR,PU,AG,CU,AC,IT,JT),(mel,mir)	0.000085	0.000060	0.000085
all-nuc	a	8	0.16	(OR,TR),(AR,MC,CN),(JR),(PU),(AG),(CU),(AC,IT),(JT),(mel),(mir)	3	0.13	(OR,TR,AR,MC,CN),(JR,PU,AG,CU,AC,IT),(JT),(mel),(mir)	0.000227	0.000151	0.000233
all-nuc	b	8	0.14	(OR,TR),(AR,MC,CN),(JR),(PU),(AG),(CU),(AC,IT),(JT),(mel),(mir)	3	0.14	(OR,TR,AR,MC,CN),(JR,PU,AG,CU,AC,IT),(JT),(mel),(mir)	0.000229	0.000159	0.000225
up-mt+nuc	a	6	0.51	(OR,TR),(AR),(MC),(CN),(IT),(JT),(mel),(mir)	2	0.22	(OR,TR,AR,MC,CN,JT),(IT),(mel),(mir)	0.000117	0.000115	0.000621
up-mt+nuc	b	6	0.57	(OR,TR),(AR),(MC),(CN),(IT),(JT),(mel),(mir)	2	0.20	(OR,TR,AR,MC,CN,JT),(IT),(mel),(mir)	0.000118	0.000119	0.000614
up-nuc	a	7	0.21	(OR),(TR),(AR),(MC),(CN),(IT),(JT),(mel),(mir)	4	0.45	(OR,TR),(AR,MC,CN),(IT),(JT),(mel),(mir)	0.000304	0.000287	0.000683
up-nuc	b	7	0.20	(OR),(TR),(AR),(MC),(CN),(IT),(JT),(mel),(mir)	4	0.44	(OR,TR),(AR,MC,CN),(IT),(JT),(mel),(mir)	0.000304	0.000282	0.000683

^a^all: all localities; up: localities upstream of the Tapajós River only; mt: sequences of mtDNA locus; nuc: sequences of nuclear loci.

^b^number of lineages retained from *Cichla pinima sensu lato*, excluding other species.

^c^PP, posterior probability of maximum *a posteriori* model.

^d^Tip arrangements (those together in parentheses) depict which tips were collapsed into a single taxon for a given collapseHeight. Locality codes follow Table 1.

^e^For relevant topological comparisons, the depth of the most recent common ancestor is listed. See text and S1 File for further explanation.

The BFD and STACEY results recognized the southern and western lineages, and potentially several nested western lineages, as exhibiting independent evolutionary trajectories consistent with the multi-species coalescent model. However, the population level data showed that these lineages were not as independent as those historical perspectives suggest, as their divergence history alone is not sufficient to explain contemporary population structure. The AMOVA of microsatellites corroborated that among the hypotheses tested above, four species (southern plus three western) was a better explanation for the data (Table 4). However, when only allele identity was considered, separating the Jari (JR) and Araguari (AR) populations explained even more of the variance in the data. Similarly, when considering allele distance, this 6 population model was superior to all hypotheses but the 4 BFD species. This demonstrates that while the four BFD species hypothesis reflects the major hierarchical pattern in the data, it obscures significant population structure among these fishes, because nested divergence with gene exchange is characteristic of this meta-population. The Structure results with *a priori* population definitions reflected a similar pattern (Fig 4, S6 Fig): under each of the 2, 3, and 4 species hypotheses, there were localities that corresponded to pure parentals as well as admixtures between the southern and western and between two western lineages. This was clear despite some continued appearance of allelic size homoplasy in the data (e.g. JR, when K = 4). Moreover, when models were tested based on simulations that should accommodate the presence of allelic homoplasy (DIYABC), models with admixture consistently exhibited higher posterior probabilities than those without admixture, for both the southern X western (0.908) and western X western (0.718) models. The rates and times of admixture were different for each downstream locality, with median ancestry estimates ranging from 6–72% (derived from Western) at ~890–4,900 generations ago (μ = 10^−4^ mut./generation) (S8 Fig). Thus, divergence and admixture at several hierarchical levels are necessary to explain the origin of the complex genetic pattern exhibited by *Cichla pinima sensu lato*.

**Table 4:** Results from Analysis of Molecular Variance (AMOVA) of *Cichla pinima sensu lato* with 11 or 9 microsatellite loci.

**AMOVA**	**11 loci (allele identity, F_ST_)**							
Percent Variation:	1sp^a^	4spM described	2sp South-West	3sp JT^b^	3sp JT-MC-AP	4sp JT/MC-AP	5sp JT/MC-AP/JR	6sp JT/MC-AP/JR/AR
Among Groups	*na*	2.19	10.28	13.64	11.33	13.94	16.22	17.41
Within Groups	25.29	24.05	19.17	16.6	17.18	15.23	13.13	11.57
Within Populations	74.71	73.76	70.55	69.76	71.49	70.83	70.64	71.02
	**9 loci (allele distance, R**_**ST**_**)**							
Percent Variation:	1sp	4sp	2sp	3sp	3sp	4sp	5sp	6sp
Among Groups	*na*	*-7*.*92*	26.37	34.57	34.79	41.02	38.38	38.55
Within Groups	25.86	54.17	29.57	22.67	20.61	15.43	16.84	15.67
Within Populations	74.14	53.75	44.06	42.77	44.6	43.55	44.78	45.78

^a^Population groupings for 1 to 4 species follow Table 2; 5 and 6 groupings build on the optimal 4-species structure.

^b^Locality codes follow Table 1.

## Discussion

### Data congruence and evolutionary units of *Cichla*

The molecular datasets from *Cichla pinima sensu lato* do not coincide with the species recognized by Kullander and Ferreira (K&F, [29]), whose results have been formalized as *Cichla pinima*, *C*. *vazzoleri*, *C*. *jariina*, and *C*. *thyrorus*. However, these datasets also initially appear to be incongruent, both internally and with one another. For example, the mitochondrial sequences portray two deeply divergent lineages that are polyphyletic within the genealogy of all *Cichla*, but were present together in at least one deme (locality). Microsatellite data, when analyzed by genetic distances, show these individuals to have a strong meta-population structure with limited gene flow between most sub-populations, while these same data also portrayed that many of these same localities retain a signature of admixture between two historically divergent lineages. Nuclear sequences reconstruct the various populations and lineages to be monophyletic with all data but polyphyletic when some unique localities are excluded, while at the same time these data reflect the independent evolution of younger lineages nested among those that are now conjoined.

The power of the strategy applied here is that each of these apparent conundrums was largely decipherable using model-based approaches that allowed testing of the apparent conflict within and between datasets. The phylogeographic analysis revealed that the admixture represented in the microsatellite data result from a recent joint colonization of the eastern Amazon by two non-sister lineages (Southern and Western) that evolved allopatrically but that apparently did not develop intrinsic reproductive isolation. The eastern populations (downstream of the Tapajós) exhibit a hybrid ancestry that manifests itself in their nuclear sequences and which is strong enough to affect a common ancestry (monophyly) for the Southern and Western lineages, while analyses of the microsatellite data show that the degree and timing of admixture differs across these sub-populations and that this has been preserved by the low rates of contemporary gene exchange between these eastern localities. Despite this extensive admixture and apparent lack of reproductive isolation, species tree analyses not only recognized the separate evolutionary history of the Southern and Western lineages even when these lineages were reconstructed as monophyletic, but also emphasized the distinction of several nested Western localities that exhibited unique mtDNA lineages. However, the mtDNA and microsatellite data also separately confirmed that at least two of these western sub-lineages (Western-core and Aripuanã) exhibit recent gene exchange.

Although these analytical results provide a cohesive narrative for these lineages in terms of the divergence, dispersal, and admixture processes that produced the current pattern, there remain conflicts among some results that reflect not incongruences in the dataset *per se*, but rather in the philosophy behind the model and its interpretation. While the analyses employing the multi-species coalescent model (MSC) emphasize the history of populations to infer separate evolutionary trajectory, they appear to largely ignore the evidence for contemporary evolutionary interdependence reflected in signatures of admixture and ongoing gene exchange. Signature of recent gene exchange or its absence is often how species hypotheses are generated for testing with the MSC, but recent studies have shown that if terminal units are improperly defined (e.g. if patterns of contemporary gene exchange to not mirror patterns of lineage ancestry), the MSC may result in misleadingly high posterior ‘speciation’ probabilities for those lineages [56]. Moreover, it appears that the MSC may often delimit population structure along with lineages most would regard as species, since the patterns of coalescence created by the two may often appear similar [67]. This is troubling, because delimitation under the MSC has been proposed as more objective and robust than traditional morphological taxonomy [47]. However, this observation partly explains recent trends for MSC-based delimitation studies to recognize more (cryptic) lineages over traditional morphological assessments [68, 69], and in many cases to support mutually exclusive sets of species for the same datasets [70–72]. Thus current implementations of the MSC model, though attractive for species delimitation, are not without subjectivity, and may deliver different results depending on how each individual would answer ‘where does meta-population structure end and divergence with gene flow begin?’, a conundrum that has its roots in the philosophy of species and not statistical accuracy.

It should go without saying that inference of population structure or species boundaries with any data type will only be as robust as the samples upon which inference is made, in particular their number and spatial distribution [15, 18, 73, 74]. In this way the analyses herein, and our previous analyses [31], should be more robust than the previous morphological review [29], since there have been examined more samples and more characters as well as a consistent use of statistical frameworks. However, there are still some unanswered questions that will require filling several gaps in sampling. For example, why do the Aripuanã and Machado samples appear closer, based on nuclear DNA (sequences and microsats), while mtDNA portrays the Jatapu as sister to the Aripuanã lineage? Incomplete lineage sorting is possible, but, all else being equal, is less likely for the haploid, maternally inherited mtDNA [75, 76], so perhaps there is an unrecognized history of mitochondrial capture. Indeed, the STACEY species tree results seem to imply cytonuclear conflict (Table 3), and the sequence of divergence events within the western sub-clade portrayed by spherical phylogeography should be interpreted with caution. Moreover, the samples obtained here for the Aripuanã, Machado, and Jatapu Rivers came from the extreme upper or lower portions of those rivers, and these samples may over- or underestimate the diversity or structure present in the remaining drainage under isolation-by-distance. Interestingly, the lower Uatumã is apparently currently inhabited by *Cichla temensis*, not *C*. *pinima sensu lato* [29], so contemporary gene flow between the upper Uatumã (including the Jatapu) and other *C*. *pinima sensu lato* populations is unlikely.

**Fig 6:**
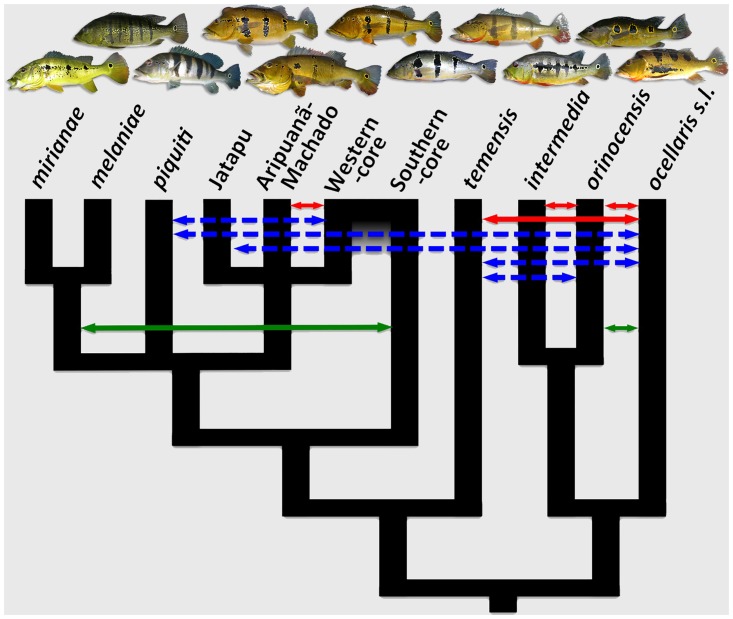
Conceptual model for the historical narrative of *Cichla*. Solid lines represent natural hybridization (red, recent; green, ancient), while blue dashed lines represent hybridization in anthropogenically altered conditions. Branch lengths are not proportional. See text for details.

Additional samples, however, while insightful into the into the processes involved in creating this diversity, can only make the current narrative more complex, not less. Fig 6 shows a conceptual model of the lineages of *Cichla* based on previous studies [31, 34, 77] and the current results (see also S9 Fig). It also shows inferred hybridization in natural and anthropogenically altered habitats [31, 78–80], which shows that incomplete reproductive isolation is common across these lineages. It does not portray the full range of morphological, ecological, or other forms of evolutionary diversity, which are key aspects of what “species”, in its general use, should capture. Nevertheless, it demonstrates that reconciliation of the evolutionary patterns of *C*. *pinima sensu lato* with all but the most arbitrary definitions of “species” cannot be done without ambiguity. Any of several renderings (one, two, or four species) would be accurate with respect to history and contemporary structure, but each would require subjective preference of criteria for conferring the species rank and would obscure the very processes that have contributed to this diversity. For example, though the Southern and Western lineages had a separate evolutionary origin (they are not monophyletic), they apparently did not develop reproductive isolation, and there are now a comparable number of admixed populations as non-admixed and they may now be functionally inseparable. On the other hand, recognizing these as a single, hybrid species would ignore the nascent lineages in the west and the ongoing processes of diversification occurring there. It would also treat each of the admixed populations as equal replicates of the parental species, despite evidence that they are unique in the degree and age of admixture, with presumably unique solutions for genome integration and local adaptation. More broadly, if the western lineages, or western and southern lineages, are synonymized because of ongoing gene exchange, historical admixture, or simply incomplete reproductive isolation, what does this imply about the remaining species of *Cichla*, which exhibit degrees of hybridization between both the closest and most distantly related species?

The answer to this question is not clear. It is clear that the four species recognized by K&F [29] do not correspond to the lineages distinguished by the analyses herein. However, these authors made a significant effort to document the morphological variation present in an important genus of Neotropical fishes, and generated significant insight. Our previous study [31] benefited from the framework created by this morphological review. However, this does not negate the fact that the morphological study failed to recognize several of the major lineages of *Cichla*, and, conversely, formalized recognition of populations that grade one into another or were indistinguishable genetically. While some of this incongruence can be attributed to smaller sample sizes, insufficient statistical framework, and a different paradigm for alpha taxonomy, I predict an equal part of the incongruence results from the ambiguity over what species are and what they are expected to represent by the many who use them.

### Are *Cichla* outliers?

The historical narrative reconstructed for *Cichla pinima sensu lato*, and *Cichla* in general, suggests that treating any hierarchical level in the data as equivalent to “species” would be arbitrary and would result in formalized ambiguity of patterns that are the essential qualities of evolutionary diversification (e.g. morphological discontinuity, independent evolutionary trajectory). It is worth asking if *Cichla* are different from other such lineages. There is a paucity of datasets of Neotropical fishes with sufficient extent to quantify both species and population-level discontinuity and test the degree to which these can be unambiguously discriminated, and none with scope similar to the dataset available for *Cichla*, but some comparisons are possible. In a molecular study of the genus *Satanoperca*, an eartheater cichlid, divergent lineages were discovered that had been previously unrecognized by morphology, while other lineages described from limited morphological data graded one into another ([81]; see also [82]). A study of the oscar cichlid, *Astronotus*, found that there was no correspondence between the two mitochondrial lineages discovered and the two morphologically-described species, *A*. *ocellatus* and *A*. *crassipinnis* [83]. A recent phylogeographic study of *Prochilodus*, the flannelmouth characins, determined that several species recognized from morphology could not be distinguished with molecular data, and this despite having relatively few samples from the widely distributed species [84]. Similar results have been found for needlefishes [85], shovelnose catfishes [86], discus cichlids [87], several groups of Central American cichlids (e.g. [88, 89]), among others. Some of these incongruences can be explained by inadequate sampling and scrutiny, and some to previous use of questionable methods to recognize species, but other cases show nested patterns of divergence and gene exchange not unlike those observed in *Cichla*.

This pattern of poor correspondence between expectations of “species” and the features of evolutionary lineages is not limited to the Neotropics or to fishes. Accumulating data from broad examinations of variation at the phylogenetic tips show that there is frequently no rectification between “species” and nested evolutionary lineages that does not require a subjective distinction, and that this pattern is common across taxa and regions (e.g. [70, 71, 90–92]). This ambiguous correspondence should be a null hypothesis for Neotropical freshwater fishes, the assumed pattern until proven otherwise with reasonable statistical confidence (e.g. [15]).

### Why do we describe species?

To be clear, this is not an issue of imperfect knowledge of the natural history or variation of a set of individuals, but rather that even under complete documentation of biological diversity there would still be disagreement among biologists as to which hierarchical divisions correspond to “species” [9]. This begs the question, ‘Is ambiguity a natural consequence of applying “species” to evolutionary phenomena?’[9]. “Species” originally and inescapably represents the least inclusive taxonomic rank [93]. The origin of describing fundamental kinds dates at least to classical Greece [94], but Linnaeus is perhaps most famous for formalizing it in Systema Naturae as evidence of The Creator’s genius [94, 95]. Most systematists now try to reconcile taxonomy, including “species”, with the structure produced by evolution [12], and since we now recognize evolution as the force generating biological diversity, this is the only justifiable reason for describing species [12, 96]; otherwise they are tantamount to baseball cards, a collection of limited intrinsic value. In this way, “species” represents a vehicle for communicating what we have discovered about evolution [12].

While a laudable and logical goal, it has never been effectively demonstrated that this reconciliation is attainable, or even fruitful [8, 97, 98]. Nonetheless, in an attempt to rectify taxonomic paradigms of “species” with conceptual models of evolutionary diversification and empirical data, de Queiroz [4] described a “general lineage concept” following definitions of “species” from early in the New Synthesis [44, 99, 100]. Recognizing that the “contingent properties” of meta-population lineages, which are the form of species in this ontology (see also [45]), may not apply to all lineages or may evolve in different orders following divergence, so the general lineage concept provides that any one property should be sufficient to support a “species hypothesis” (sensu [101]). It is unclear how, under this paradigm, it is possible to conclude that two groups are NOT different species (i.e. resolution of data conflicts), since the basic notion of a hypothesis is that it is disprovable [102]. Nonetheless, the description aptly captures what most biologists now understand to be the basic nature of evolutionary diversity.

While this proposition has considerable merit, it also has a fundamental problem: “species” is a rank in a hierarchy, not an affirmation that there is hierarchical structure in nature, and it is impractical to use the term “species” and not conflate these two [96]. The hallmarks of the species rank are formality and exclusivity: species are described using formal rules and an individual or population cannot reside in multiple ranked species simultaneously. Formality is mutually exclusive with ‘species are hypotheses’ since it implies certainty, discourages re-examination, and leads non-systematists to assume that all named lineages will have the same properties, including reliability. Similarly while named species are invariably conceptualized as exclusive (dichotomous) branches, this appears frequently not to be the case, rendering application of the species rank arbitrary (not unlike other taxonomic ranks [94]).

### Future directions

It is ultimately impractical to expect that the species rank can be equated with evolutionary phenomena given mounting evidence of the arbitrary way in which this must be done [9]. In this way, “species” is a red herring: something that serves to confuse and distract from the goal, which is to document and understand evolutionary processes [13]. Instead, researchers should focus on quantifying the diversity in nature, at whatever hierarchical level it occurs, rather than applying labels that are counterproductive to our goals, and work to interpret what that variation means about contemporaneous and historical processes that contributed to that diversity [2, 8, 9].

It will be argued that eliminating “species” from scientific use would be detrimental to those disciplines that rely on species. On the contrary, the consequences of species ambiguity in ecology, biogeography, and conservation have been well described, including “taxonomic inflation” and the incorrect assumptions of equivalence inherent in formal description [6, 9, 103–106]. In particular, shifting taxonomic decisions away from biological reality towards conservation goals risks eroding the perception of neutrality and objectivity that scientists must be afforded by policy makers in order for our results to be heeded [9, 107]. It would be naïve to expect that we will cease to use labels to refer to evolutionary phenomena, since some labels serve as a convenient shorthand to describe meta-population lineages (e.g. Western and Southern) [2], but we should do so without the confusion that applying a formal rank or label necessarily creates.

Although proposing a system to refer to evolutionary phenomena as an alternative to “species” is beyond the scope of this paper, it would seem less ambiguous, and thus ultimately more efficient, for studies using lineages as the basis of their work to include informal verbal descriptions of these meta-population lineages accompanied by visual aids (photos, maps, phylogenies, frequency distribution plots, etc.) to describe the units under study. Indeed, this is what most studies already do (e.g. [108]), and the transition of the peer-reviewed literature to digital media makes this more feasible. Ultimately, each study should be responsible for explicitly describing the methods by which the units of analyses are defined for peer review, in the way that other methods are subject to review, which is predicated on concerted efforts by those who discover biodiversity to make appropriate data available to the scientific community to use in this manner. Moreover, that we will be breaking with tradition is not a reason to continue to rely on a vehicle that poorly captures the phenomena that we are trying to convey, especially when there must be more useful alternatives.

Although the analyses used here focus largely on molecular data, other forms of data have a valid place in quantifying population variance and evolutionary divergence. However, species lineages, insofar as they exist, are emergent properties of population-level evolutionary processes [2]. While many data types can be used to decipher these processes, they are inherently genetic phenomena, heritable changes in populations being the definition of evolution [109]. Thus, there is a natural logic to using genetic data to investigate the past and present effects of the forces of evolution (mutation, selection, drift, gene flow) (e.g. [110]). Moreover, as the current data demonstrate, not having a broad understanding of the phylogeographic history of a species can lead to erroneous estimates of phylogenetic relationships or distinctiveness depending on which samples are chosen [111], information not generally recoverable with morphological data. Nonetheless, phenotypic data can and should play an important role in describing population variation, but should also be subjected to the same statistical rigors as other data types [15, 20, 112]. In particular, the paradigm of type series should receive further review, since the limited number of specimens applied, and therefore variation captured, is destined to be inadequate to describe an entire, continually evolving population, a phenomenon recognized in genetic data and elsewhere as ‘high-grading bias’ [113].

## Conclusions

The historical narrative for the populations of *Cichla pinima sensu lato* is more complex than can be portrayed by recognizing them as one, two, or four species: their history and contemporary dynamics cannot be unambiguously rendered as discrete units (taxa) at any level without both choosing the supremacy of one delimitation criterion and obscuring the very information that provides insight into the diversification process. This calls into question the utility of species as a rank, term, or concept, and suggests that while biologists may have a reasonable grasp of the structure of evolution, our methods of conveying these insights need re-evaluation. The lack of correspondence between evolutionary phenomena and discrete species should serve as a null hypothesis, and observed patterns of discontinuity should be subject to the same statistical rigor across data types. Rather than advocate for the primacy of molecules over morphology, larger samples sizes and more data of all kinds are needed.

In the present case, it seems unlikely for additional samples to make the correspondence of these populations to “species” less ambiguous, and additional data are more likely to depict the natural history of these lineages as even more complex than we currently understand. Nonetheless, these are the insights by which we may learn how diversity arises and changes. The question for Neotropical freshwater fishes and other biota should not be ‘what are the species?,’ but rather, ‘what are the patterns of variation and what do they tell us about evolutionary diversification?’ Evolutionary lineages are real, but hope for “species” to communicate these phenomena is misplaced [98]; application of “species” to lineages is too frequently arbitrary and counterproductive.

## Supporting information

S1 Fig Morphological variation in *Cichla*. Photos by the author and also provided by K. Winemiller, C. Montaña, and P. Reiss.
(TIF)

S2 Fig Fully annotated mtDNA phylogeny of *Cichla* clade A. An abridged version of this phylogeny appears in Fig 2.
(TIF)

S3 Fig Models and posterior probability for western X southern from approximate Bayesian computation in DIYABC using 9 microsatellite loci. (TIF)

S4 Fig Models and posterior probability for western X western from approximate Bayesian computation in DIYABC using 9 microsatellite loci. (TIF)

S5 Fig Ancestral distributions of the mtDNA clades of *Cichla pinima sensu lato* predicted by spherical phylogeography in Beast. Arrows in the first four panels represent the 95% highest posterior density estimates of geographic distribution for the mtDNA clades. Shaded areas represent the inferred drainage distributions of those clades. In the last panel, arrows represent the boundaries of the 95% HPD for all haplotypes in that clade. Values in each panel represent the mean and 95% HPD for the divergence (dispersal) times of the included clades (see Fig 2).
(TIF)

S6 Fig Likelihood and delta K from structure for different values of K with 11 or 9 microsatellite loci. (TIF)

S7 Fig Structure results for 9 microsatellite loci from *Cichla pinima sensu lato*. Pop = 0, no sample pre-assignment; Pop = 1, some samples pre-assigned to K clusters, indicated by the bars above the Structure plots. The distribution of mtDNA clades (after Fig 1) is provided for reference. Locality codes follow Table 1 and Fig 1.
(TIF)

S8 Fig Plots of posterior distribution from DIYABC for (A) admixture proportion and (B) admixture time of populations in the southern X western model using nine microsatellite loci from *Cichla pinima sensu lato*. In both panels, boxes represent the 25^th^ and 75^th^ percentile, while the whiskers show the 95% highest posterior density; black dots represent the mode.
(TIF)

S9 Fig Map of the distributions of the four lineages of *Cichla pinima sensu lato* recovered by BFD with sequence data, and the history of lineage admixture confirmed with mtDNA and microsatellite analyses. Color corresponds to the clusters in Fig 4 (K = 4), with purple representing the hybrid population between the southern and western core regions.
(TIF)

S1 Table Characteristics of the microsatellite data from *Cichla pinima sensu lato*. (XLSX)

S2 Table Characteristics of the sequence data from *Cichla pinima sensu lato* and congeners *C*. *piquiti* and *C*. *temensis*. S, number of variable sites. (XLSX)

S3 Table Genetic distances among localities of *Cichla pinima sensu lato* for microsatellites: Below diagonal, F_ST_ (11 loci); above diagonal, R_ST_ (9 loci). Values significant after correction for multiple tests (Benjamini-Hochberg) are shown in gray.
(XLSX)

S4 Table Posterior probability for cluster numbers from Structurama for 11 and 9 microsatellite loci. (XLSX)

S5 Table Results from runs of STACEY with different priors as well as different data arrangements and different collapseHeight summary values excluding the Machado locality. (XLSX)

S1 File Supplementary methods. (DOCX)
