# Supplementary figures and images for: One species or four? Yes!...and, no. Or, arbitrary assignment of lineages to species obscures the diversification processes of Neotropical fishes
Source: PLoS One. 2017 Feb 24;12(2):e0172349. doi: 10.1371/journal.pone.0172349 (PMC5325279; doi:10.1371/journal.pone.0172349)

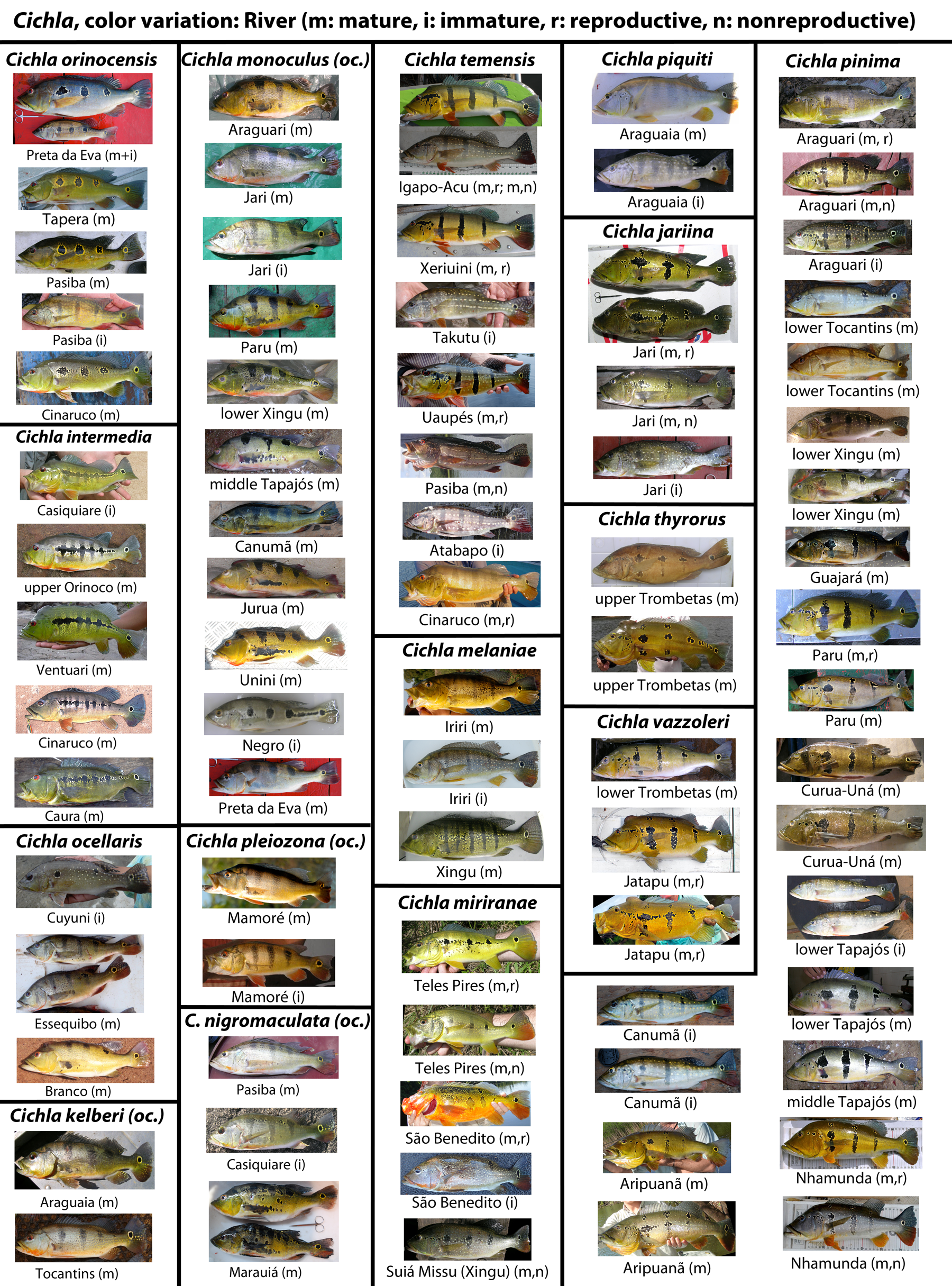

Supplement: S1 Fig — Photos by the author and also provided by K. Winemiller, C. Montaña, and P. Reiss. (TIF) [file pone.0172349.s001.tif]

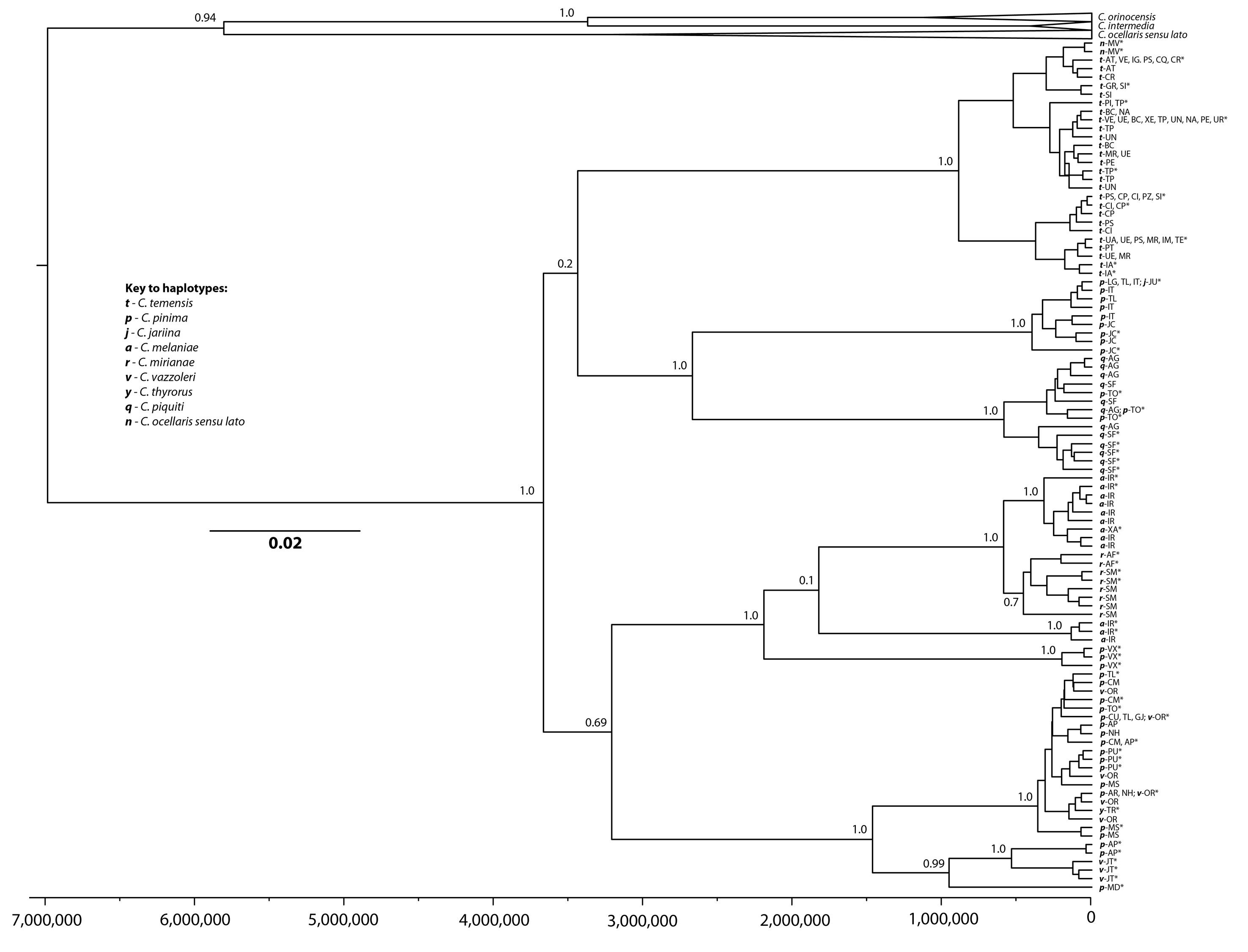

Supplement: S2 Fig — An abridged version of this phylogeny appears in Fig 2. (TIF) [file pone.0172349.s002.tif]

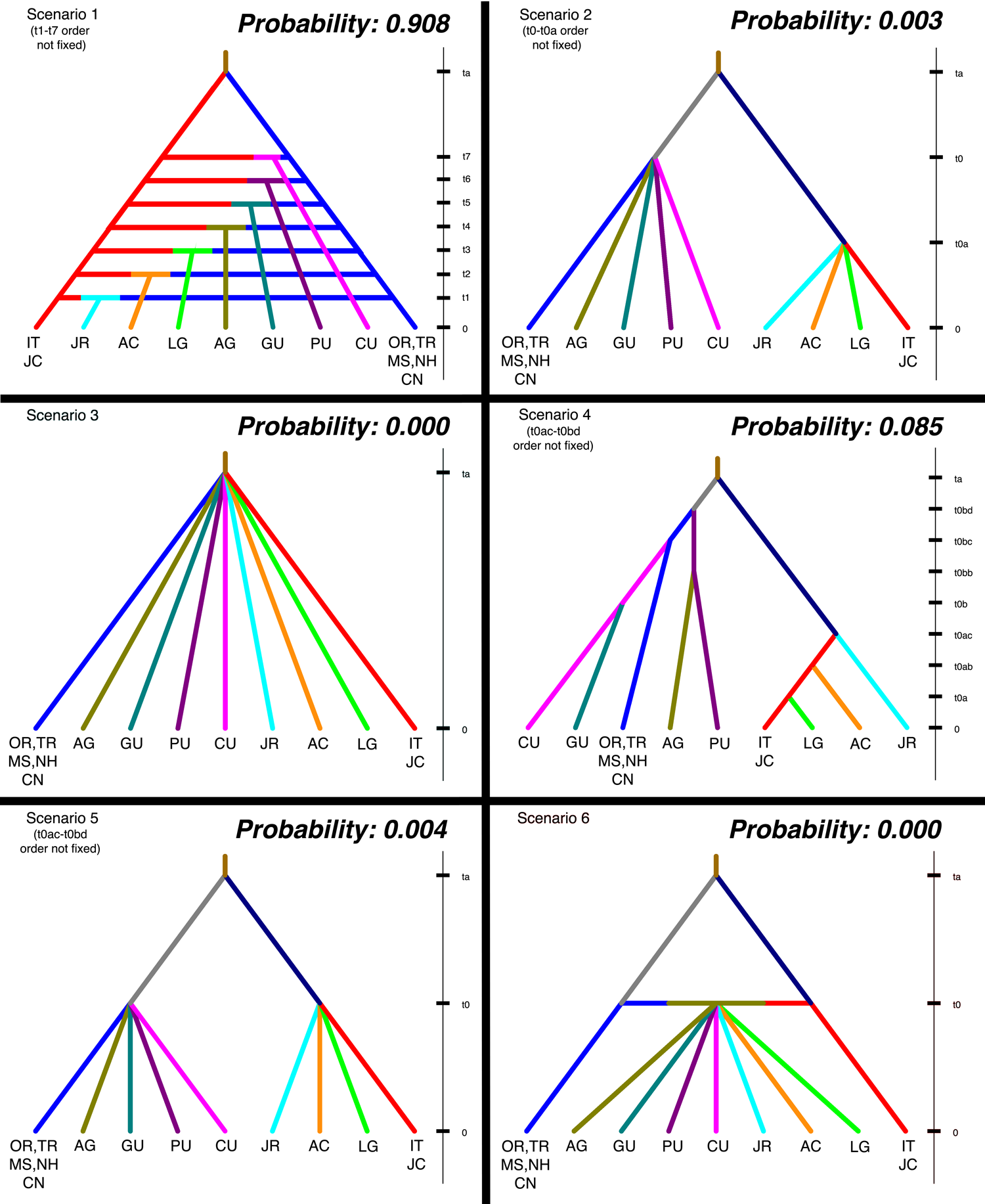

Supplement: S3 Fig — (TIF) [file pone.0172349.s003.tif]

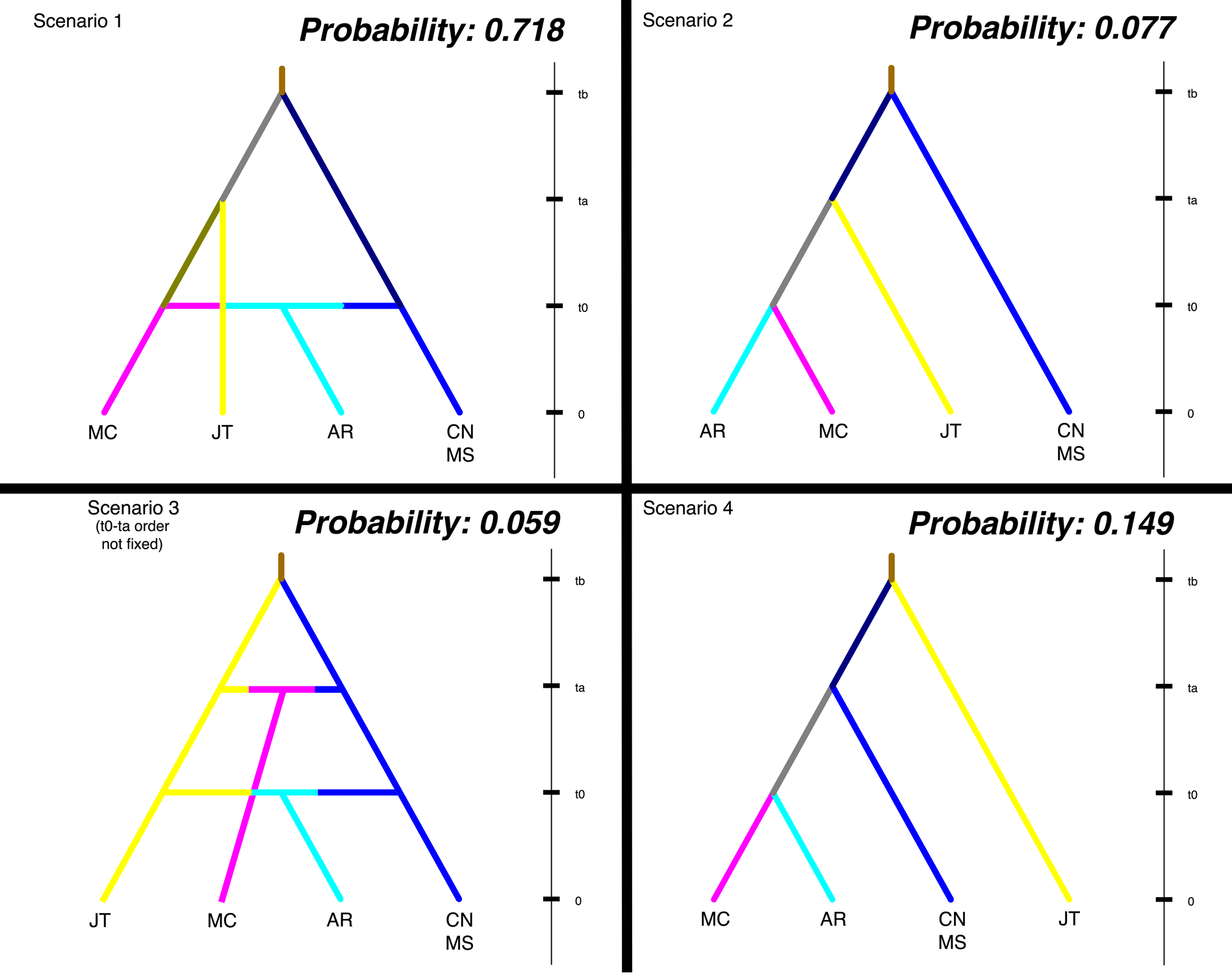

Supplement: S4 Fig — (TIF) [file pone.0172349.s004.tif]

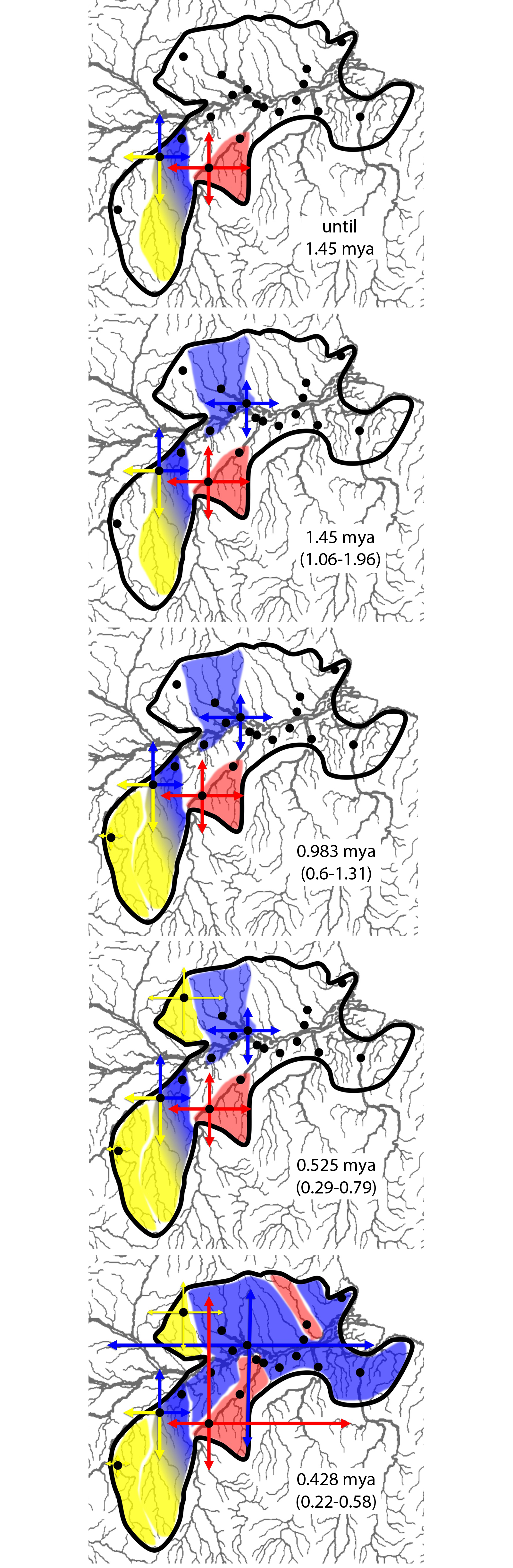

Supplement: S5 Fig — Arrows in the first four panels represent the 95% highest posterior density estimates of geographic distribution for the mtDNA clades. Shaded areas represent the inferred drainage distributions of those clades. In the last panel, arrows represent the boundaries of the 95% HPD for all haplotypes in that clade. Values in each panel represent the mean and 95% HPD for the divergence (dispersal) times of the included clades (see Fig 2). (TIF) [file pone.0172349.s005.tif]

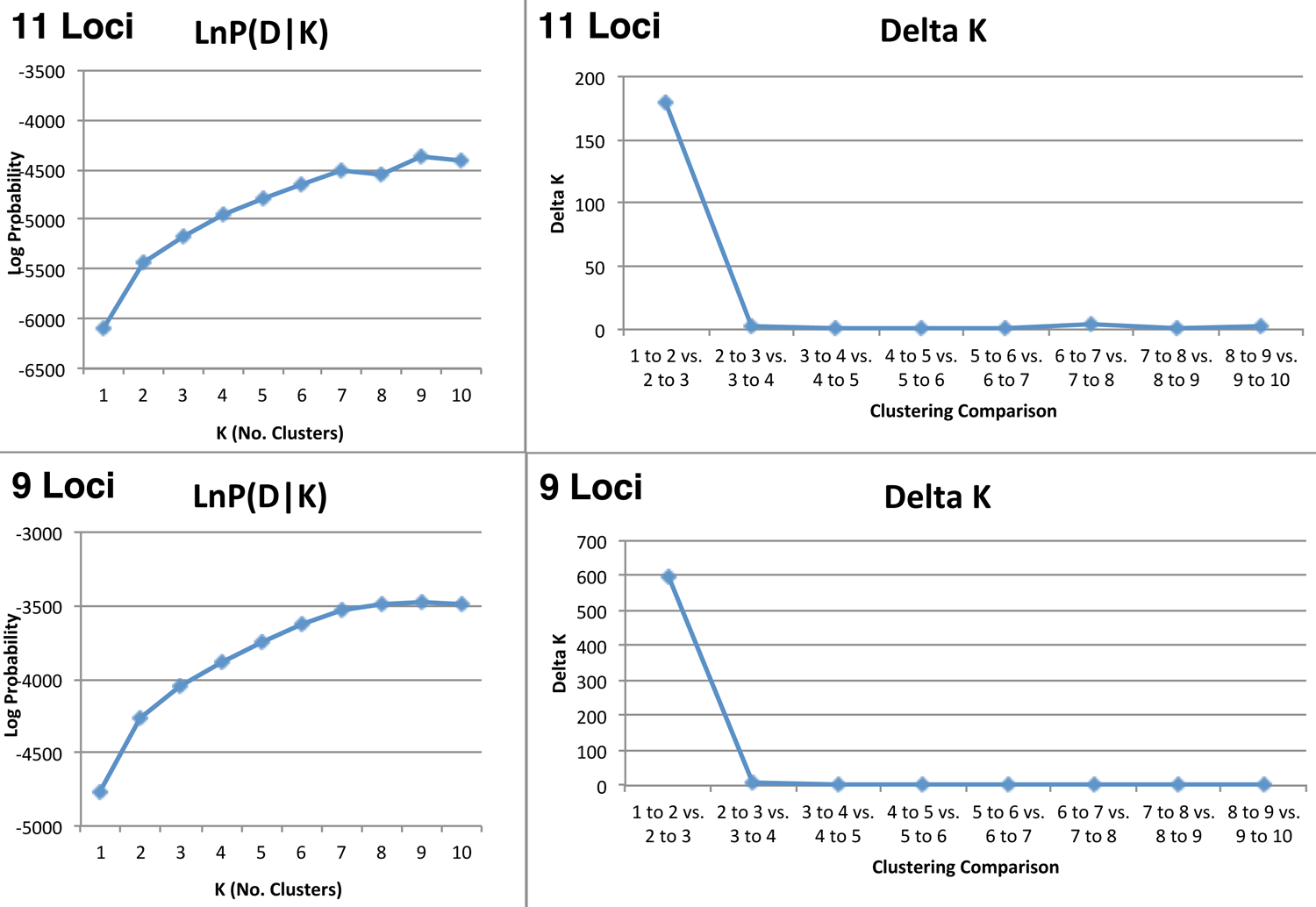

Supplement: S6 Fig — (TIF) [file pone.0172349.s006.tif]

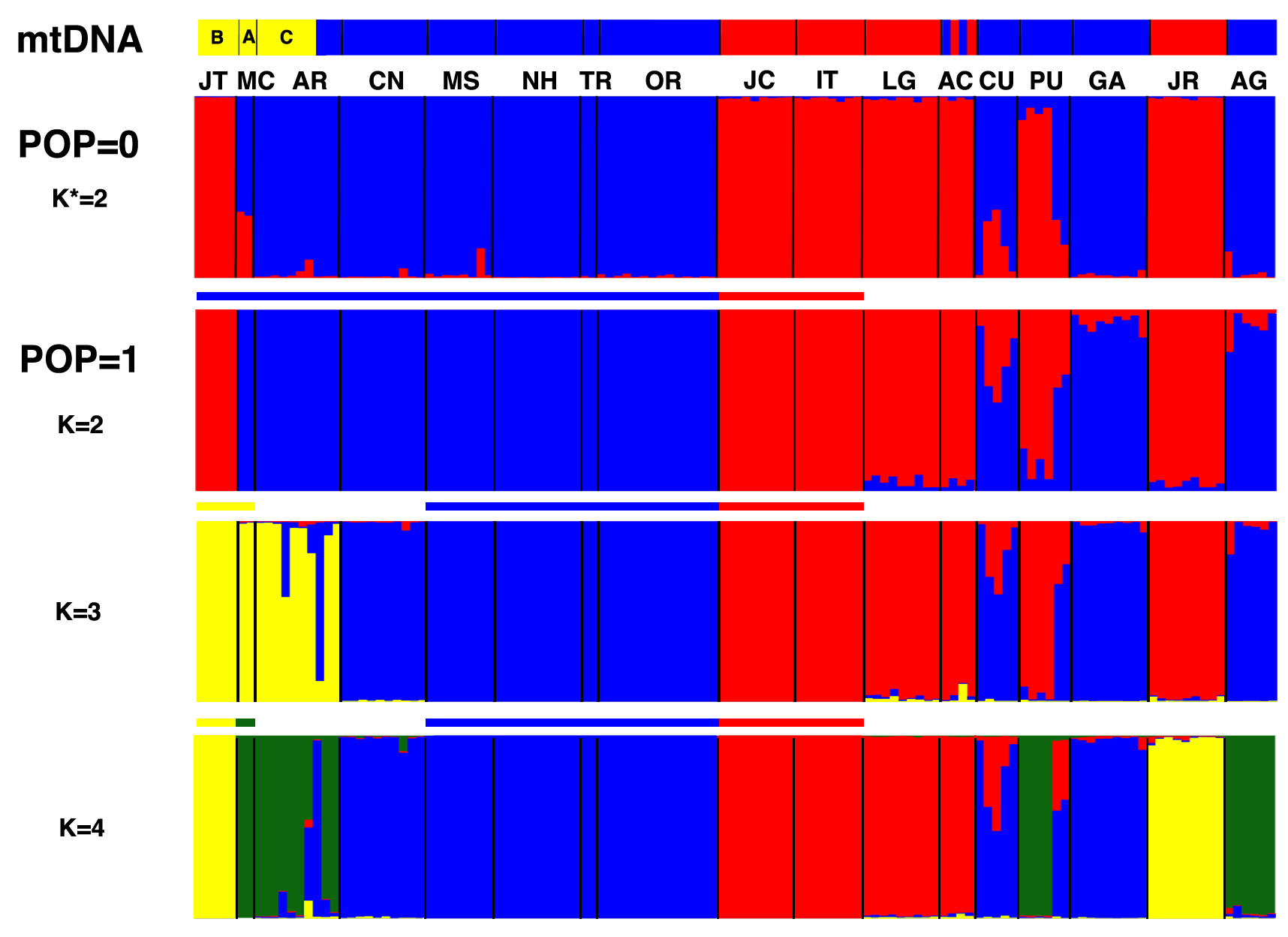

Supplement: S7 Fig — Pop = 0, no sample pre-assignment; Pop = 1, some samples pre-assigned to K clusters, indicated by the bars above the Structure plots. The distribution of mtDNA clades (after Fig 1) is provided for reference. Locality codes follow Table 1 and Fig 1. (TIF) [file pone.0172349.s007.tif]

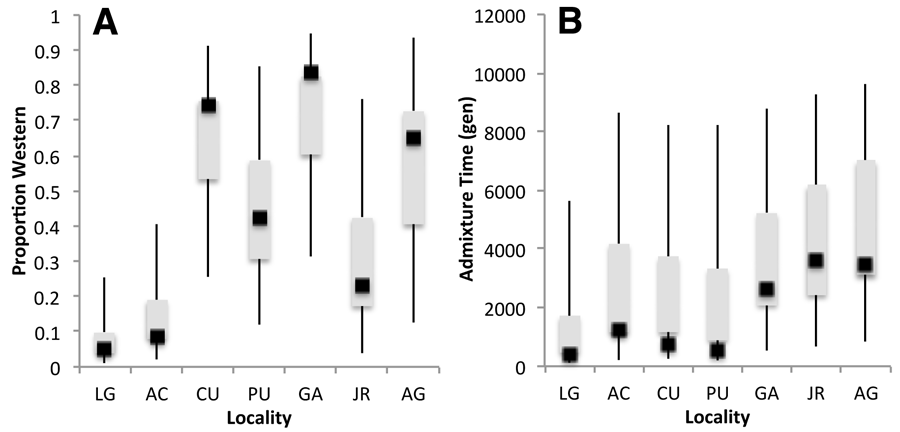

Supplement: S8 Fig — In both panels, boxes represent the 25th and 75th percentile, while the whiskers show the 95% highest posterior density; black dots represent the mode. (TIF) [file pone.0172349.s008.tif]

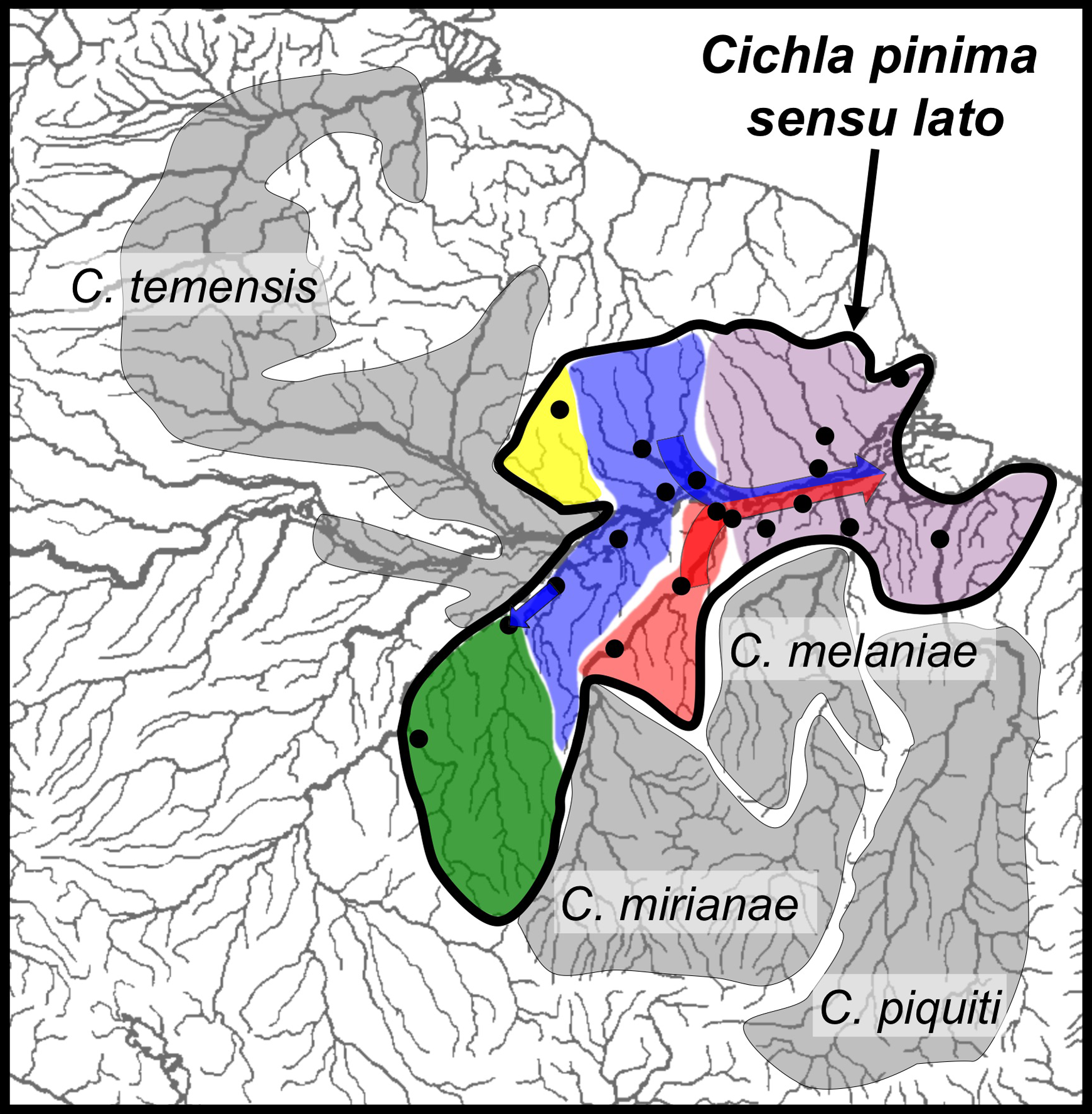

Supplement: S9 Fig — Color corresponds to the clusters in Fig 4 (K = 4), with purple representing the hybrid population between the southern and western core regions. (TIF) [file pone.0172349.s009.tif]
