# Supplementary material for: One species or four? Yes!...and, no. Or, arbitrary assignment of lineages to species obscures the diversification processes of Neotropical fishes
Source: PLoS One. 2017 Feb 24;12(2):e0172349. doi: 10.1371/journal.pone.0172349 (PMC5325279; doi:10.1371/journal.pone.0172349)
Supplement: S1 File — (DOCX) [file pone.0172349.s015.docx]

Sequence Arrangement and Model Choice

Although it has recently been shown that recombination may have little effect on species tree analyses (Lanier), a test for recombination was performed for each locus using Topali (REF). A single locus tested positive, and was reduced to the largest fragment without a signal of recombination.

Appropriate substitution models were chosen for each locus, or each gene X codon in the case of ATPase 8,6, using the Decision Theory criterion implemented in jModelTest (Posada). Models are available upon request.

MtDNA Phylogeography

MtDNA sequence haplotypes (CR, ATP) from all *Cichla* samples were analyzed in Beast 2.4 using the chosen models (2 ATP genes x 3 codons plus CR = 7 partitions) with relaxed, lognormal molecular clocks for each partition. Two identical chains of 50 million generations, sampling every 5,000 steps. The first half were discarded as burn-in, convergence and mixing were assessed using Tracer v1.6 (Rambaut & Drummond 2007), and the two posterior samples were combined. The tree was calibrated using a mutation rate of 0.02 mutations/site/million years, comparable to 1% per million years in cytochrome b (Willis 2006). This rate is consistent with a rate for African cichlids (Genner et al. 2007).

Spherical phylogeography was performed using two partitions for only the mtDNA data from *C. pinima sensu lato* (CR, ATP), using sequences from every individual (alleles) except those from the Xingu and Tocantins (see main text). Branch lengths were modeled with strict molecular clocks, and a Bayesian skyline prior. Two runs consisting of 500 million generations were made, sampling every 50,000 generations, and discarding the first 50% as burn-in. Ancestral distributions were estimated by summary with TreeAnnotator (part of the Beast suite).

Population Structure with Microsatellites

Expectations of Hardy-Weinberg equilibrium for each locus in each locality was tested using G_IS_ in Genodive v2.0b27 (Meirmans & Van Tienderen 2004); only a single locus (C11) at a single locality (Maues, MS) was found to deviate from expectations after correction for multiple tests (sequential Bonferroni), and no correction was made. Whether the size of alleles reflected additional population structure over identity-based distances was tested in Spagedi 1.4b using 1,000 permutations. Structure was run 20 times for each K from 1 to 10, each run consisting of 100,000 iterations for burn-in and posterior. To compare results with Structure, Structurama was run 5 times for each prior value of 2, 5, and 10 clusters, for 100,000 generations each time, sampling frequency of 100, with 20 heated chains with a temperature of 0.03. Of these, 50% of samples were discarded as burn-in. The posterior probability of each K was estimated by summing the posterior frequency of K across all runs (S. Table 3).

Bayes Factor Delimitation using *Beast and Path Sampling

Units emphasized by the phylogeography and population structure analyses provide preliminary hypotheses of species units to be tested by Bayes Factors using the implementation of the multi-species coalescent in *Beast. To compare the different species models, the likelihood of the species tree was inferred for several species groupings, from one to four species, using the *Beast model and path sampling in Beast 2.4. This analysis used sequences from 17 of the 21 nuclear loci from Willis et al. (2013), as well as mtDNA (ATPase) data; five nuclear loci were left out because they showed no variation in *Cichla pinima sensu lato* (SI Table 2). Analyses were run with several dataset variations. First, species models were tested with and without mtDNA, since mtDNA may provide a biased impression of population structure and trans-specific gene exchange (Funk & Omland). Second, models were also run without data from localities downstream of the Tapajós River mouth (or Lago Grande), since these showed admixture between the two major clusters, and as such are inconsistent with the model’s assumptions of no post-divergence gene flow.

For evaluation of the described species hypothesis, individuals (alleles) were assigned to species based on locality origin and assignment in Kullander & Ferreira (2006). For the remaining models, individuals from each locality were assigned to hypothesized species units according to a neighbor-joining phenogram of possession of shared alleles distance at 11 microsatellite loci inferred using Microsatellite Analyzer (Dieringer & Schlötterer 2003) and Phylip 3.6 (Felsenstein), depicted below.


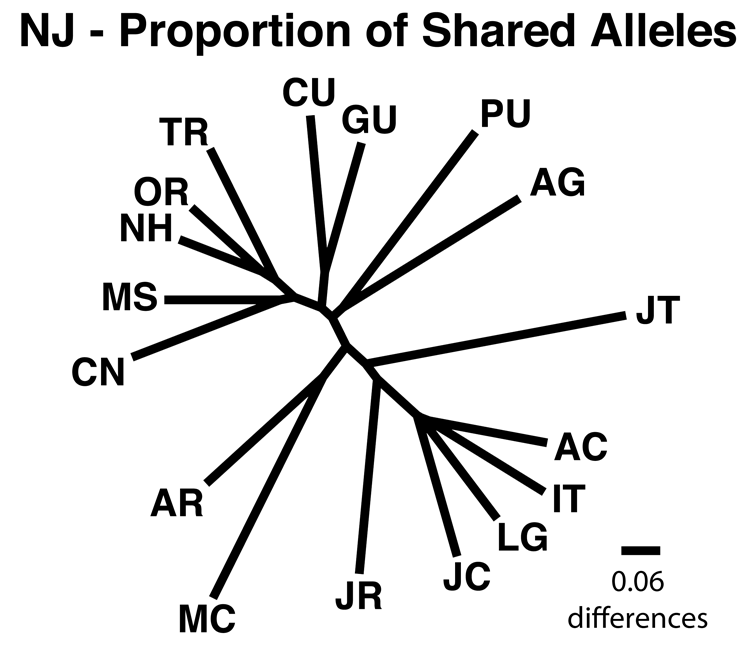


For each of a total of 3 dataset versions (with and without mtDNA data, and with mtDNA but without any data from downstream localities;), and for each of the arrangements of “species” depicted in Table 3, XML files were created for Beast 2.4 using the *Beast template in Beauti. Mutation models were those chosen by jModelTest (DT), and each of the loci (separate gene tree partitions) received a strict molecular clock except for each of the 6 partitions for ATP (mtDNA, which had a linked gene tree), which was modeled with a relaxed, lognormal clock. The appropriateness of strict clocks was previously tested (Willis et al. 2013). The rate prior for the clock for each nuclear partition was given a normal distribution with a mean and standard deviation of 1 (relative to the first nuclear locus) and hard bounds of 0 to 10, while the 6 partitions of ATP were modeled with uniform distributions with upper bound of 150. The popMean parameter was given an exponential distribution with mean of 1x10^-4^, and was modeled on each branch with a linear with constant root model. The Yule birthrate was modeled with a uniform distribution with upper bound of 10,000.

Each XML was run for 600 million generations to achieve convergence (assessed using ESS values in Tracer); this took ~3-4 days on a Dell high performance workstation depending on the dataset. Into the XML files were then inserted commands for path sampling (following the tutorial on http://beast2.org), which consisted of 48 steps with 100 million generations in each step and an alpha of 0.3, and commands executed in Beast to create shell scripts to run divide steps into sets to run sequentially while each set ran simultaneously (“threads” equaled 24 sets of 2, or 16 sets of 3). The post-burin-in xml.state file was used to start each set of steps (24 or 16) and then 10% of each step was discarded as burn-in prior to estimating the marginal likelihood. Each 100 million generation step, run on a separate processor of a Dell workstation, took ~6-12 hours to complete depending on the dataset. ESS values were assessed for each step and those with ESS less than 50 were run for additional iterations of 100 million generations until the ESS achieved this level.

Bayesian Phylogeny and Phylogeography (BPP)

BPP is another implementation of the multi-species coalescent that allows for gene tree discordance in the species tree model via a reversible-jump MCMC search, but only allows sequence data to be modeled according to the Jukes-Cantor substitution model (Yang & Rannala 2010). The following parameters were used to estimate population sizes (theta) and branch lengths (mutations/site) for the optimal Bayes Factor Delimitation species tree hypothesis, from which were calculated coalescent branch lengths (N_E_ generations) in order to predict the expected rates of gene tree discordance in clade A. Each run consisted of 30,000 samples collected at a frequency of every 5 generations (total 150,000 generations) after a burn-in of 60,000 generations using the a(0,0) algorithm (fixed species tree; only branch lengths and population sizes were optimized). Four runs were made with different priors for population size and root depths that corresponded to total tree depths of 0.2, 2, 8, and 20 coalescent units (according to the formula BL/(theta/2); Degnan & Rosenberg 2009). Exact prior specifications are available upon request. Data used in this analysis included mtDNA and nuclear data from the non-downstream *C. pinima sensu lato* (see text), *C. piquiti*, and *C. temensis*. Individual loci were specified to have different relative mutation rates estimated from the *Beast analyses above. Coalescent branch length for clade A were calculated using the inferred root depth and mean theta for the extant lineages.

STACEY

A second implementation of the multi-species coalescent model to test the fit of this model to different number of species is in the package STACEY (Graham et al.), also implemented in Beast 2.4. In this analysis, the terminal units are minimal units that may be species, generally demes or other homogenous populations, and a species tree is inferred among these tips. The novel part of STACEY (in addition to new tree search operators) is the application of a birth-death-collapse tree prior, a modification of the traditional birth-death prior in which branches estimated to be shorter than a specified length (collapse height) are collapsed to a single branch (based also on the collapse weight prior). The length of the branches in the species tree, of course, depend on the population size, so the prior on this parameter (popScale) can be important as well. I made four arrangements of the data, similar to the above: with and without mtDNA (ATP), and with and without the downstream data. Tips in this analysis were localities of *Cichla pinima sensu lato* for which data from all loci were available; otherwise, species were coded as delimited by Willis et al. (2012). I also made data arrangements that included the Machado (MC) locality, which exhibits a unique mtDNA lineage, but for which only data of the mtDNA and two most variable nuclear loci were available; to code this as a separate tip, I included dummy sequences with missing data for the remaining 15 nuclear loci. To determine the effects of the aforementioned priors, I also ran several iterations of the first dataset with different values for these priors.

Input XML files for Beast 2.4 for the STACEY analysis were also prepared with Beauti with the appropriate template. Datasets were similar to above except that 1) terminal units within *C. pinima sensu lato* were localities, and 2) all four combinations of with/without mtDNA and with/without the downstream localities were run, as well as with and without the Machado locality (which only exhibited two nuclear genes, mitf and xsrc, plus ATP). To speed convergence, topology constraints were imposed on the species tree (but not gene trees) to enforce the monophyly of clades A and B (Willis et al. 2006), and for *C. temensis* to be sister to the remaining clade A species. Both of these topologies are recovered 100% in the maximum clade credibility tree of unconstrained species tree analyses (above). Mutation models and rates were the same as above. Each of 2 runs consisted of 200 million generations, sampling every 5,000 generations and discarding 50% as burn-in; convergence was assessed using ESS in Tracer. To include the Machado locality, a single “all missing data” sequence was inserted into each of the remaining 15 genes; this appeared to have no impact on convergence time, ESS, or collapse results. In all runs except those where the effect of each prior was tested (S. Table 4), popPriorScale was given an exponential distribution with mean of 1x10^-4^, collapseWeight prior was given a beta distribution with alpha/beta of 1/1 (uniform probability for numbers of terminal “species” branches), and the collapseHeight prior was 1x10^-5^. After 50% burn-in, results from each run were summarized with collapseHeights of 1x10^-5^ (more species) and 1x10^-4^ (fewer species). The number of remaining tips (“species”) and posterior probability for this model was recorded for both collapseHeights for each run.

Corroborating Multi-species Coalescent with Microsatellite Data

Analysis of molecular variance (AMOVA) was performed on the 11 and 9-locus microsatellite datasets, testing allele identity with the first set, and allele distance in the second. This was performed in the program Arlequin 3.5, and tested with 1,000 permutations. Assignment probability and mixture proportions using *a priori* assignment of the core localities from the hypothesized species lineages to pre-defined clusters (POP-INFO) was performed in Structure for both the 11 and 9 locus datasets. Runs consisted of 100,000 generations with equal burn-in.

Different hypotheses for how the downstream and lower Madeira localities came to show their contemporary patterns were tested by comparing different scenarios of population history using approximate Bayesian computation in the program DIYABC. Six models were constructed for the Southern X Western comparison (Supplemental Figure 3), and four for the western-only data (Supplemental Figure 4). These models were tested with the 9-locus microsatellite dataset with motif-conforming allele sizes that could be effectively modeled with a generalized stepwise mutation model (S. Table 2). The global mutation rate was fixed at 10^-4^ mutations per generation, while rates among loci were not fixed. Except in cases where it was unavoidable due to topology, the order of events was not fixed in each model. Priors for population size and divergence/admixture times were chosen based on estimates from runs of Migrate-n (Beerli 2006) and preliminary runs of DIYABC (available upon request); appropriateness of these priors was estimated by principal components analysis of simulated vectors of population statistics and the observed vector (below). Model probabilities were estimated by simulating 10 million vectors and calculating the logistic probability from the 100,000 closest vectors. For the chosen model for the Southern X Western scenario, 20 million vectors were simulated for this model alone to make posterior estimates of the admixture rates and times from the closest 2,000 vectors.


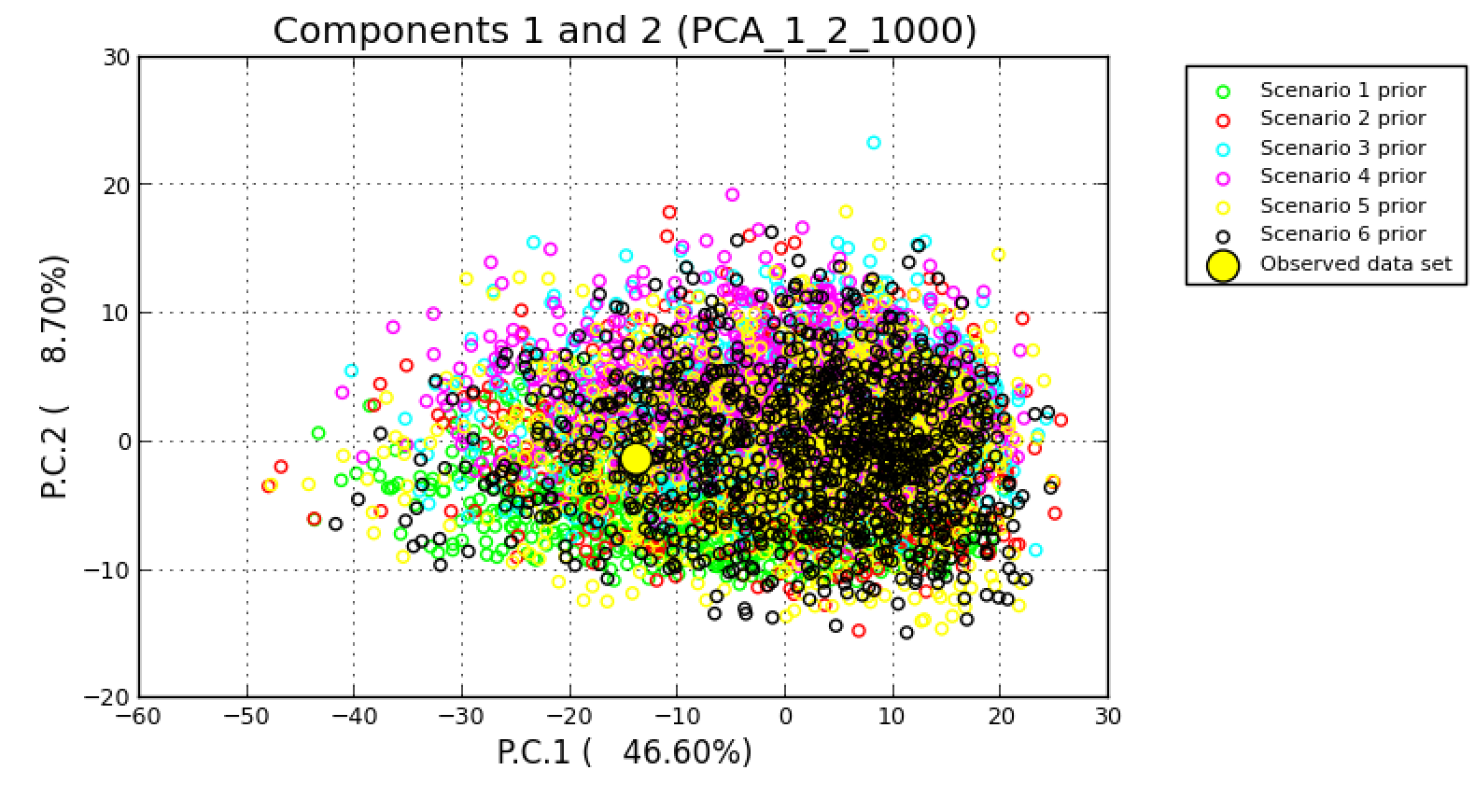

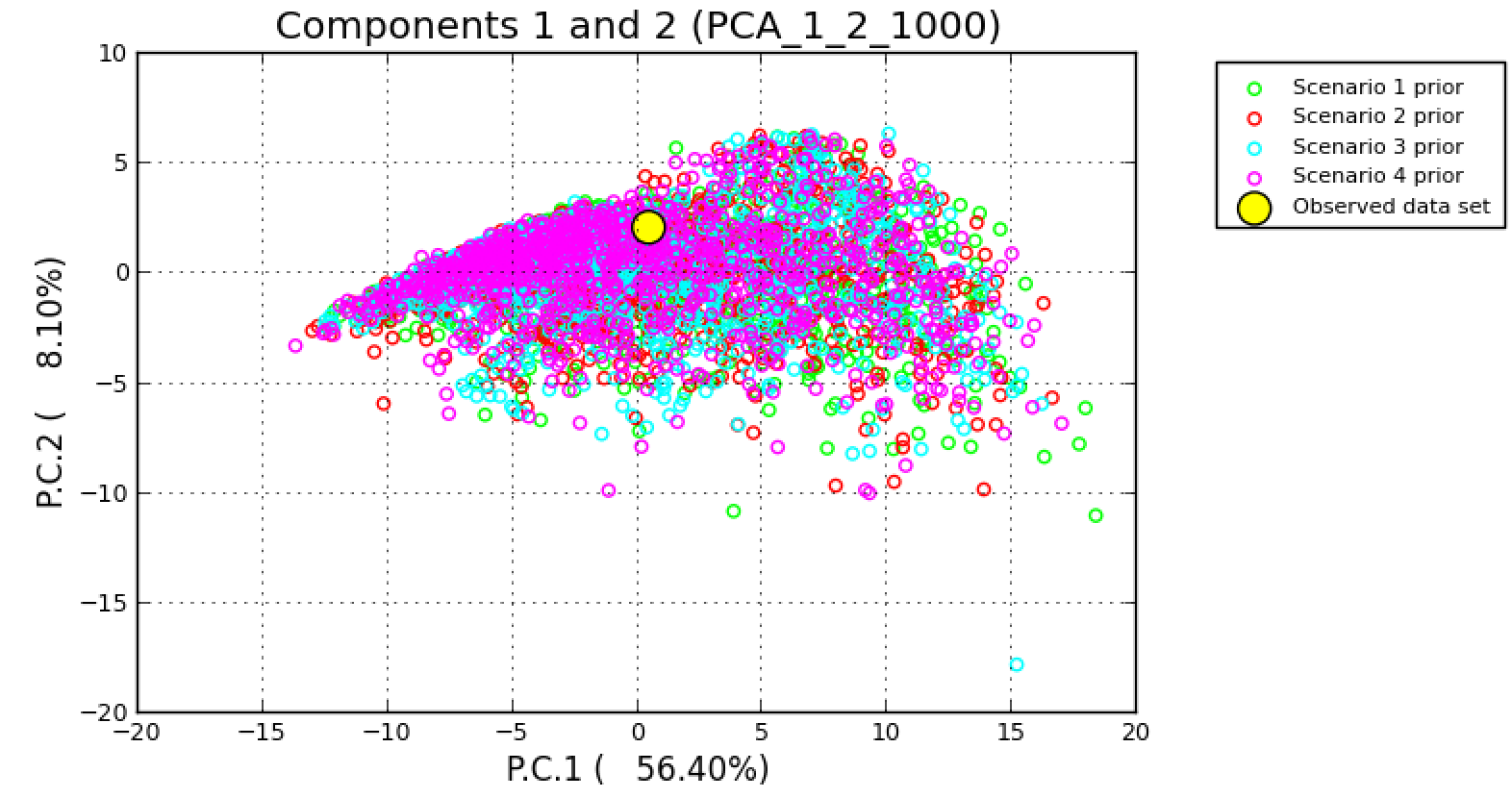


References cited only in the Supplemental Information

Beerli P (2006) Comparison of Bayesian and maximum likelihood inference of population genetic parameters. *Bioinformatics* **22**, 341-345.

Dieringer D, Schlötterer C (2003) Microsatellite analyser (MSA): a platform independent analysis tool for large microsatellite data sets. *Molecular Ecology Notes* **3**, 167-169.

Felsenstein J (2005) PHYLIP (Phylogeny Inference Package) version 3.6. Distributed by the author., Seattle.

Meirmans PG, Van Tienderen PH (2004) GENOTYPE and GENODIVE: two programs for the analysis of genetic diversity of asexual organisms. *Molecular Ecology Notes* **4**, 792-794.

Rambaut A, Drummond AJ (2007) Tracer v1.4. Available from <http://beast.bio.ed.ac.uk/Tracer>.
